# CryoSIP: unleashing protein high-resolution Cryo-EM via semantic-instance collaborative picking

**DOI:** 10.1093/bib/bbag138

**Published:** 2026-04-01

**Authors:** Yu Deng, Shengxiang Wang, Mingrong Xiang, Yuxin Li, Linlin Zhuo, Dongsheng Cao, Xiangzheng Fu, Quan Zou

**Affiliations:** Engineering Research Center of Polyploid Fish Reproduction and Breeding of the State Education Ministry, College of Life Sciences, Hunan Normal University, Changsha, Hunan 410081, PR China; Yuelushan Laboratory, Changsha, Hunan 410128, PR China; State Key Laboratory of Developmental Biology of Freshwater Fish, Hunan Normal University, Changsha, Hunan 410081, PR China; School of Data Science and Artificial Intelligence, Wenzhou University of Technology, No. 337 Jinhai Avenue Wenzhou, Zhejiang 325027, China; School of Data Science and Artificial Intelligence, Wenzhou University of Technology, No. 337 Jinhai Avenue Wenzhou, Zhejiang 325027, China; School of Data Science and Artificial Intelligence, Wenzhou University of Technology, No. 337 Jinhai Avenue Wenzhou, Zhejiang 325027, China; School of Data Science and Artificial Intelligence, Wenzhou University of Technology, No. 337 Jinhai Avenue Wenzhou, Zhejiang 325027, China; Xiangya School of Pharmaceutical Sciences, Central South University, No. 87 Xiangya Road, Changsha, Hunan 410003, China; School of Information Engineering, Changsha Medical University, No. 1501 Lei Feng Avenue, Changsha 410219, China; College of Computer Science and Electronic Engineering, Hunan University, No. 2 Lushan South Road, Changsha 410012, China; Institute of Fundamental and Frontier Sciences, University of Electronic Science and Technology of China, No. 1 Chengdian Road, Minjiang Avenue, Chengdu 611730, China

**Keywords:** particle picking, cryo-electron microscopy (cryo-EM), protein 3D reconstruction, multi-frequency adaptive U-net framework, semantic-instance collaborative picking

## Abstract

Precise particle picking in cryo-electron microscopy (cryo-EM) single-particle analysis constitutes a fundamental challenge in achieving high-resolution 3D reconstruction. To address low detection rates and elevated false positives caused by low signal-to-noise ratios (SNRs) and weak image contrast, we developed a novel semantic-instance collaborative picking framework. Key innovations include: (i) A multi-frequency adaptive U-Net framework that precisely localizes particles via global-context semantic modeling and multi-scale feature fusion; (ii) CryoSIP further enhances the collaborative optimization between SAM and U-Net by refining the interaction between SAM and U-Net–derived semantic priors, thereby improving instance mask generation. Experiments demonstrate that our framework reduces false positives significantly compared to state-of-the-art tools while achieving high recall in particle picking. 3D reconstructions using this approach exhibit improved density map resolution, demonstrating its potential for advancing atomic-resolution cryo-EM structural analysis.

## Introduction

Cryo-EM has emerged as a transformative technology in structural biology. It reshapes the traditional paradigm of structural analysis by enabling near-atomic resolution in 3D reconstructions [1]. In contrast to X-ray crystallography, which relies on well-ordered crystals, cryo-EM uses rapid freezing to preserve the native structures of biological macromolecules—such as dynamic protein complexes and viral particles—in near-physiological conditions, thereby eliminating the reliance on static crystals [2]. This ‘resolution revolution’ not only bridges gaps in the structural analysis of flexible and heterogeneous macromolecules, but also advances mechanistic understanding of dynamic molecular machines [3]. However, accurate localization of individual particles—the cornerstone of 3D reconstruction—remains challenged by low SNR (e.g. <0.5) and complex imaging artifacts, which significantly impair the accuracy of atomic-level model building [4, 5].

As a critical preprocessing step in cryo-EM 3D reconstruction, the reliability of single-particle localization directly influences the accuracy of atomic model construction. Traditional approaches fall into two categories: (i) Manual annotation, which may consume up to 70% of the total processing time for 3000 × 3000 pixel images, suffers from significant variability in particle boundary definitions due to operator subjectivity; (ii) Semi-automated methods—including template matching (e.g. RELION [6], EMAN2 [7]) and geometric feature detection (e.g. DoG Picker [8], XMIPP [9], FindEM [10])—enhance throughput but are constrained by template biases. When structural differences between the initial template and target particles are substantial, recognition accuracy declines sharply [11]. Existing methods often exhibit high false positive rates when applied to low-SNR images (e.g. <0.5). Moreover, these methods are prone to missed detections when identifying heterogeneous particles with substantial conformational variability, potentially leading to pronounced local resolution variations in the reconstructed density map [12].

With the rapid advancement of deep learning, convolutional neural networks (CNNs) have been increasingly adopted for particle picking, leading to the development of various model architectures, including Topaz [13], crYOLO [14] (based on the YOLO object detection framework [15]), and CASSPER [16] (employing semantic segmentation). Subsequent methods, including DRPnet [17], APPLE Picker [18], Deep Picker [19], DeepCryoPicker [20], and Warp [21], have enhanced automation in particle detection and localization by advancing feature extraction and pattern recognition strategies. Despite notable progress, current methods still encounter two major bottlenecks: (i) High false positive rates in low SNR (e.g. <0.5) images, primarily due to misidentification induced by background noise and contaminants; (ii) Substantially reduced recognition accuracy for particles exhibiting size heterogeneity (>30% variation) and conformational diversity (e.g. multi-subunit dynamic assemblies), highlighting the limitations of existing algorithms in feature space modeling.

The success of visual Transformers in natural language processing has catalyzed their adoption in computational microscopy, particularly in cryo-EM image analysis, due to their powerful global attention mechanisms. Unlike traditional CNNs, Transformers capture long-range dependencies at the pixel level via multi-head self-attention, enabling effective modeling of subnanometer morphological differences and densely packed protein distributions. Transformer-based frameworks such as Detection Transformer (DETR) [22] explicitly model spatial constraints between particles by integrating target queries with global contextual encoding—an essential capability for addressing low SNR and blurred boundaries typical in cryo-EM images. Leveraging these advantages, CryoTransformer [23] introduced a multi-scale attention fusion architecture, demonstrating strong performance across multiple benchmark datasets. Compared to traditional CNN-based methods, it significantly improved both protein particle detection accuracy and the resolution of reconstructed density maps, underscoring the strong potential of Transformer architectures for high-precision 3D cryo-EM reconstruction.

Net [24], a milestone architecture in medical image analysis, has been highly successful in cryo-EM particle segmentation due to its symmetric encoder-decoder structure and cross-layer skip connections [25]. Its core strength lies in multi-scale feature fusion, which captures the global distribution of particles through four levels of downsampling (max pooling). Additionally, it preserves details and progressively restores particle boundary features using upsampling convolutional layers. Recently, Meta AI’s Segment Anything Model (SAM) [26] has emerged as a powerful general-purpose segmentation model, demonstrating remarkable zero-shot and few-shot transfer learning capabilities across various image domains. Its ability to automatically segment arbitrary objects opens new possibilities for robust particle picking. CryoSegNet [25] employs a two-stage strategy: first, using U-Net [24] for particle segmentation and then applying SAM for boundary recognition. By combining these two stages, more particles can be collected. However, this method still faces issues with edge blurring and misclassification of heterogeneity.

To address this, the study proposes a protein particle picking scheme based on a semantic-instance collaborative mechanism, aiming to achieve high-resolution 3D protein reconstruction. First, in the preprocessing stage, the unsupervised Zero-Shot Noise2Noise (ZS-N2N) framework is employed for structure-preserving denoising [27], enhancing feature fidelity in low signal-to-noise ratio regions and collaborating with anisotropic Gaussian filtering to suppress background noise, thereby providing high-fidelity input for the downstream segmentation network. Secondly, a multi-frequency domain adaptive U-Net architecture is constructed, incorporating the Pyramid Vision Transformer v2 (PVTv2) encoder [28] to capture multi-scale global context features. Simultaneously, the decoder’s multi-frequency domain edge representation capability is enhanced through the wavelet convolution operator [29], ensuring accurate positioning of protein particle regions. Additionally, a semantically guided instance-adaptive mechanism is designed to collaboratively extract features using U-Net semantic segmentation and SAM instance detection, improving boundary clarity and reducing the false positive rate. Building on this, the study enhances the accuracy of protein particle picking and advances high-resolution 3D protein reconstruction. This study uses the CryoPPP dataset [30], sourced from the Electron Microscopy Public Image Archive (EMPIAR) [31]. A 22-protein training set was used to train the model, while seven independent [32–38] protein test sets were employed to systematically evaluate the model’s accuracy, robustness, and reconstruction consistency. The method’s applicability in real cryo-EM scenarios was verified from multiple perspectives.

This study proposes a protein particle picking framework based on a semantic–instance collaborative mechanism to enable high-resolution 3D protein reconstruction. First, during preprocessing, an unsupervised framework is employed to perform structure-preserving denoising and enhance feature fidelity in low SNR regions. Additionally, anisotropic Gaussian filtering is applied to suppress background noise and provide high-fidelity input to the downstream segmentation network. Second, a multi-frequency domain adaptive U-Net is constructed, incorporating a pyramid vision encoder to extract multi-scale global context features. Simultaneously, wavelet convolution is used to enhance the decoder’s edge representation in the multi-frequency domain, enabling precise localization of protein particle regions. In addition, we refine the U-Net–SAM collaborative optimization proposed in CryoSegNet [25] by introducing an additional interaction between U-Net and SAM to improve final instance masks and reduce false positives. Building on these components, the framework improves the accuracy of protein particle picking and advances high-resolution 3D protein reconstruction. The model is trained on 22 protein datasets and evaluated using seven independent test sets to systematically assess its performance in particle picking and 3D reconstruction.

## Results

### Model operation framework

**Figure 1:**
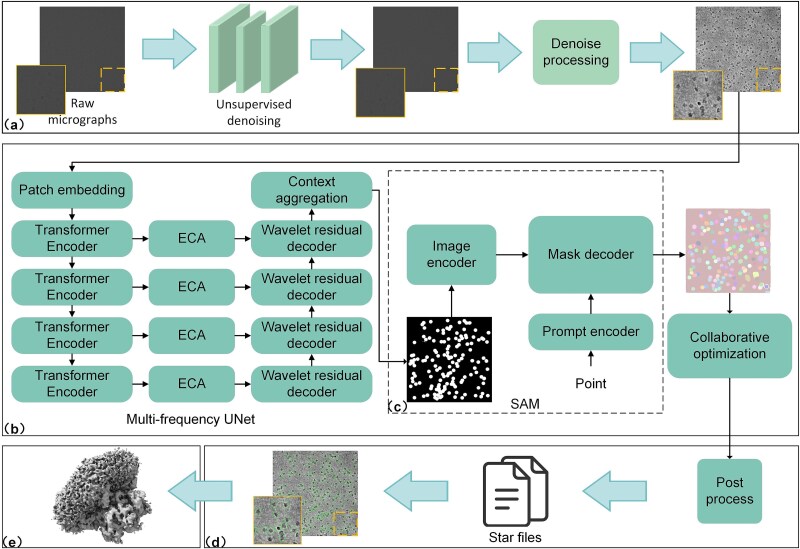
Overview of the CryoSIP workflow. CryoSIP facilitates protein particle picking and high-resolution 3D structure reconstruction from cryo-EM images. The workflow consists of five main stages. (a) Image preprocessing: A combination of unsupervised and traditional denoising techniques is applied to enhance contrast and improve the signal-to-noise ratios (SNR), thereby increasing the visibility of particles in raw cryo-EM images. (b) Multi-frequency domain U-Net: The encoder, based on Pyramid Vision Transformer v2 (PVTv2), captures multi-scale global contextual features. The decoder integrates wavelet transforms and residual connections to enhance edge representation and multi-scale feature reconstruction. The ECA module improves semantic consistency across skip connections, enabling precise semantic segmentation of protein particles. (c) SAM-based instance segmentation: The segment anything model (SAM) refines preliminary particle mask boundaries produced by the multi-frequency domain U-Net. (d) Semantic-instance co-optimization: This stage integrates coarse-grained semantic segmentation with fine-grained instance detection to refine particle masks. Candidate instances are filtered based on spatial overlap, morphological consistency, and prediction confidence. High-confidence particles are retained to ensure semantic consistency and precise boundary localization. Final particle coordinates and radii are stored in the standard .star format for downstream processing. (e) High-resolution 3D reconstruction: Optimized particle coordinates and .star files are input into CryoSPARC to reconstruct high-resolution protein structures.

Figure 1 illustrates the operational workflow of CryoSIP, comprising: (a) image preprocessing, (b) multi-frequency domain adaptive U-Net architecture, (c) SAM-based instance segmentation, (d) semantic–instance collaborative optimization mechanism, and (e) high-resolution 3D reconstruction of protein structures. In module (a), denoising methods [27, 39–41] are applied to the original cryo-EM protein images to enhance contrast and SNR while preserving particle fidelity. Note that the raw microscopic image exhibits inherently low contrast. For better visualization, the image contrast is enhanced. The detailed image preprocessing procedure is provided in Supplementary Fig. S1. In module (b), the PVTv2 encoder captures multi-scale global contextual features, the decoder’s edge representation in the multi-frequency domain is enhanced via a wavelet convolution operator, and a gated attention mechanism (ECA) improves semantic consistency in skip connections, enabling accurate semantic segmentation of protein particles. In module (c), SAM is applied to the semantic segmentation output from module (b) to perform instance segmentation and delineate protein particle boundaries. In module (d), outputs from semantic segmentation (module b) and instance segmentation (module c) are jointly optimized to achieve high-precision protein particle picking. In module (e), the refined protein particle images are integrated, and CryoSPARC [42] is employed to reconstruct the high-resolution 3D protein structure.

This study evaluates the performance of the proposed CryoSIP particle picking framework using the CryoPPP dataset [30]. The training set consists of 22 protein datasets, while the test set includes 7 independent protein image datasets, representing various particle morphologies, background complexities, and imaging qualities. Detailed dataset information is provided in Supplementary Tables S4 and S5; definitions of the gold standard, positive and negative samples, and the experimental settings are described in Supplementary Notes 2 and 3. Additionally, high-resolution 3D protein reconstruction is performed based on the results of particle picking. The main objectives of the experiment are to address three research questions.

- RQ1: Is the proposed protein particle picking model effective?
- RQ2: Does accurate protein particle picking contribute to high-resolution 3D protein reconstruction?
- RQ3: Do the key design components contribute to both protein particle picking and high-resolution 3D protein reconstruction?

### Semantic-instance collaborative optimization enables accurate protein particle picking (RQ1)

**Figure 2:**
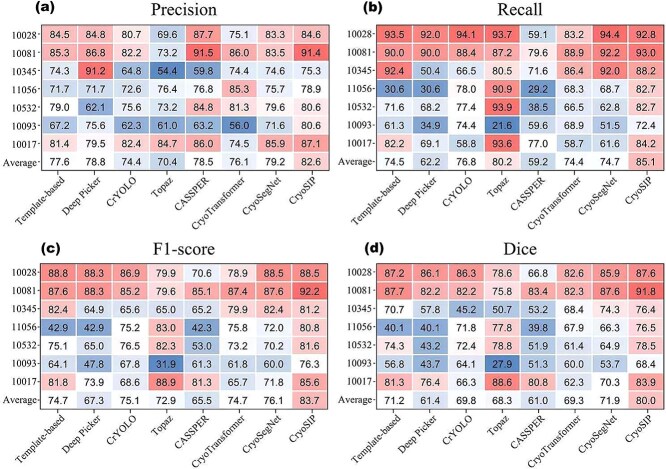
Comparison of protein particle picking performance across multiple cryo-EM datasets (EMPIAR-10028, −10081, −10345, −11056, −10093, −10017) using various methods, evaluated by (a) precision, (b) recall, (c) F1-score, and (d) dice coefficient.

To evaluate the performance advantage of CryoSIP in protein particle picking, we conducted a systematic assessment on seven unseen protein datasets, comparing it with mainstream methods including Topaz, CrYOLO, CryoTransformer, and CASSPER. Results in Fig. 2 indicate that CryoSIP achieved the highest performance across key metrics, with an F1-score of 83.7%, precision of 82.6%, recall of 85.1%, and a Dice coefficient of 80%. Notably, on the Transport dataset, CryoSIP achieved an F1-score of 92.2%, precision of 91.4%, and recall of 93.0%, outperforming methods such as Topaz and CASSPER. Notably, on the Transport dataset, CryoSIP achieved an F1-score of 92.2%, precision of 91.4%, and recall of 93.0%, outperforming methods such as Topaz and CASSPER. This balance and robustness stem from the integrated semantic–instance coordination mechanism and noise suppression strategy, which effectively mitigate the precision–recall trade-off observed in traditional methods.

Further analysis reveals that existing methods exhibit clear limitations across different scenarios. For instance, although Topaz achieves a high recall of 80.2%, it suffers from low precision (70.4%) and a high false positive rate. CASSPER exhibits high precision (78.5%) but insufficient recall (59.2%), making it unsuitable for primary screening tasks. Methods like Deep Picker and CrYOLO demonstrate large performance fluctuations across datasets and limited generalizability. The box plot in Supplementary Fig. S2 further quantifies this instability: Topaz and CryoTransformer exhibit extreme precision outliers on certain datasets, with values as low as 0.4. CryoSIP shows a median precision above 75.0%, a narrow interquartile range, and no observed outliers. Its minimum Recall (72.0%) is also markedly higher than that of CASSPER (29.2%) and Deep Picker (30.6%), demonstrating superior robustness and consistency.

CryoSIP demonstrates stable generalization performance under diverse data conditions. On EMPIAR-10081 (126,000 particles), its recall reaches 82.7%, outperforming CASSPER (29.2%) by a large margin. Furthermore, CryoSIP maintains high recall while significantly improving precision. For instance, CryoSIP’s recall approaches Topaz’s 90.9%, with a relative precision improvement of 5.7%. On the low-contrast Signaling dataset, CryoSIP achieves an F1-score of 81.2%, substantially outperforming Deep Picker (64.9%). These advantages stem from its multi-level denoising process and dynamic feature weighting mechanism, which enhance adaptability to complex backgrounds. Table 1 further confirms that particles selected by CryoSIP yield higher resolution in subsequent 3D reconstructions, underscoring its practical value in real-world structural analysis.

**Table 1:** Resolution of protein 3D reconstruction using different particle picking methods (Å).

EMPIAR ID/models	Template-based	Deep Picker	CrYOLO	Topaz	CASSPER	CryoTransformer	CryoSegNet	CryoSIP
10,028	4.12	4.09	4.11	3.97	4.43	3.86	4.18	4.13
10,081	5.26	6.05	5.38	5.10	5.79	5.47	5.71	3.81
10,345	4.07	9.13	3.94	3.66	5.14	6.43	5.71	3.79
11,056	8.12	9.65	8.54	8.06	8.47	7.42	7.23	5.11
10,532	3.93	4.92	4.10	4.27	3.96	3.92	4.08	3.80
10,093	5.85	7.50	6.93	6.15	7.27	6.86	7.12	3.96
10,017	5.00	5.63	4.87	5.09	5.33	5.61	6.83	4.71
Average	5.19	6.71	5.41	5.19	5.77	5.65	5.83	4.19

To reduce potential data leakage and more fairly evaluate generalization, we decreased train–test sequence similarity using MMseqs2 (https://github.com/soedinglab/MMseqs2) with a 30% sequence-identity cutoff: any test entry identical to, or with identity >30% to, a training sequence was removed. This analysis revealed that EMPIAR-10345 in the test set matches EMPIAR-10096 in the training set; accordingly, we excluded EMPIAR-10345 when recomputing averages. The results in Table 2 show that our method remains best on all averaged metrics after this exclusion, further supporting its effectiveness and robustness.

**Table 2:** Average performance of protein particle picking performance across multiple cryo-EM datasets (EMPIAR-10028, −10081, −10345, −11056, −10093, −10017) using various methods. EMPIAR-10345 was excluded from the test set due to its high similarity to the training data. M1–M8 correspond to the template-based, deep picker, CrYOLO, Topaz, CASSPER, CryoTransformer, CryoSegNet, and CryoSIP models, respectively.

Models/Metrics	M1	M2	M3	M4	M5	M6	M7	M8
Precision	0.782	0.768	0.760	0.730	0.817	0.764	0.799	0.839
Recall	0.715	0.641	0.785	0.802	0.572	0.724	0.719	0.846
F1-Score	0.734	0.677	0.767	0.743	0.656	0.738	0.750	0.842
Dice Score	0.712	0.620	0.739	0.713	0.623	0.694	0.715	0.811

Additionally, we added a inference efficiency comparison in covering CrYOLO, Topaz, CryoTransformer, CryoSegNet, and our CryoSIP. The inference time and memory usage reported in Table 3 were evaluated using 100 images randomly selected from EMPIAR-10081. Inference time and memory usage were measured as described below. Inference time was defined as the total elapsed time from reading the raw image, including image preprocessing (uniform resizing to 1024 × 1024), forward inference of the backbone network, mask binarization, and subsequent refinement using the SAM automatic mask generator. The reported inference time corresponds to the average over 100 images. GPU memory usage was measured using the *torch.cuda.max_memory_allocated*() interface provided by PyTorch. Memory statistics were reset before each inference, and the peak memory consumption during a single inference run was recorded (in MB). The maximum value across the 100 runs was reported. All experiments were conducted on a server equipped with an NVIDIA GeForce RTX 4090 GPU and a 16-vCPU Intel Xeon Platinum 8352 V processor (2.10 GHz). During inference, *torch.no_grad*() was enabled to disable gradient computation, and the image preprocessing pipeline was fixed to ensure stable and reproducible results. Table 3 reports throughput and peak memory under matched hardware/software settings. CryoSIP surpasses CryoSegNet in both inference speed and memory usage, and its memory footprint is comparable to CrYOLO and Topaz. Although CrYOLO achieves the lowest latency, its accuracy in low-contrast regions is markedly lower; by contrast, CryoSIP delivers substantial efficiency gains while maintaining high accuracy. These results indicate that CryoSIP offers a favorable accuracy-efficiency trade-off for high-throughput applications.

**Table 3:** Comparison of inference efficiency across models (runtime and GPU memory).

Models/Metrics	CrYOLO	Topaz	CryoTransformer	CryoSegNet	CryoSIP
Inference Time	0.30s	2.32 s	3.09 s	8.10s	5.08 s
Inference GPU Mem	5.31G	4.45G	2.15G	12.0G	5.84G

### Does accurate particle picking enhance high-resolution protein 3D reconstruction (RQ2)

The CryoSIP method significantly enhances the resolution of 3D reconstructions. As shown in Table 1, the CryoSIP method demonstrates significant advantages in most protein reconstruction scenarios, as indicated by the 3D reconstruction resolution of the EMPIAR dataset. Its average resolution reaches 4.19 Å, significantly outperforming other methods, including Deep Picker (6.71 Å) and CryoSegNet (4.98 Å). Among the seven datasets, CryoSIP achieved the best resolution in five of them. For instance, on the Transport dataset (EMPIAR-10081), which contains a large number of particles, CryoSIP achieved a resolution of 3.81 Å, more than 1.2 Å higher than Topaz (5.10 Å) and CASSPER (5.79 Å). On the low-contrast Signaling dataset (EMPIAR-10345), CryoSIP achieved a resolution of 3.79 Å, significantly outperforming Deep Picker (9.13 Å) and CryoTransformer (6.43 Å). This outcome is directly correlated with the exceptional performance of CryoSIP in precision (82.6%) and recall (85.1%). CryoSIP’s precise particle positioning reduces false detection noise, while its high recall rate ensures the inclusion of complete particle information, resulting in a higher-resolution density map in 3D reconstruction.

The resolution limitations of traditional methods are closely tied to their performance. Compared to other methods, Topaz has a higher recall (80.2%), but its low precision (70.4%) leads to false particle detection, which interferes with reconstruction quality. For instance, on the EMPIAR-11056 dataset, Topaz’s resolution (8.06 Å) is significantly lower than CryoSIP’s (5.11 Å). CASSPER missed key particles due to low recall (59.2%), resulting in a resolution (5.77 Å) more than 1.6 Å lower than CryoSIP. Deep Picker and CryoSegNet exhibit significant performance fluctuations. Deep Picker achieves a resolution of 9.13 Å in EMPIAR-10345, likely due to insufficient effective particles, with a recall of only 50.4%. CryoSegNet achieves a resolution of 4.08 Å in EMPIAR-10532, but its resolution sharply drops to 7.12 Å and 6.83 Å in other datasets (e.g. EMPIAR-10093 and EMPIAR-10017), indicating that the algorithm lacks robustness. These findings confirm the strong correlation between particle detection accuracy and 3D reconstruction quality. CryoSIP has achieved a breakthrough in high-resolution 3D protein reconstruction through balanced detection capabilities (F1 = 83.7%) and stable spatial positioning (Dice = 80.0%).

**Figure 3:**
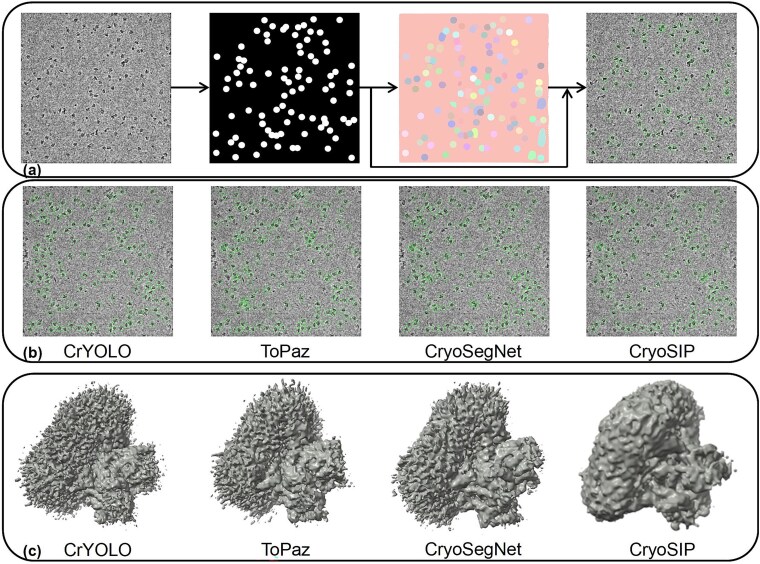
Performance comparison of CryoSIP and other segmentation algorithms (CrYOLO, ToPaz, CryoSegNet) on the EMPIAR-10081 dataset, covering protein particle detection and 3D reconstruction in three parts: (a) the transformation from input to output using the CryoSIP method: the first sub-image shows the denoised input, the second and third sub-images display the multi-frequency U-Net and SAM segmentation results, and the fourth sub-image presents the particle picking result after collaborative screening. (b) Protein particle detection results for the four methods (CrYOLO, ToPaz, CryoSegNet, CryoSIP) on the EMPIAR-10081 dataset, with green circles indicating the detected protein particles. (c) A comparison of the 3D reconstruction results of the four methods on the EMPIAR-10081 dataset.

Visualization of high-resolution 3D protein reconstructions. In the visualization comparison of the EMPIAR-10081 dataset, the proposed method demonstrates significant advantages in particle picking and 3D reconstruction. As shown in Fig. 3a, the multi-frequency domain semantic segmentation U-Net is combined with SAM instance segmentation to accurately pick protein particles. In the first stage, a coarse-grained mask is generated using multi-frequency U-Net to capture the global distribution of particles. In the second stage, the SAM model is applied to perform adaptive boundary optimization, accurately identifying particle contours through a cross-stage feature collaboration mechanism (as indicated by the arrow). The two-stage collaboration significantly enhances segmentation robustness in complex scenes, particularly in low signal-to-noise ratio areas (dashed boxes). SAM’s fine-grained segmentation capability effectively suppresses background interference, while multi-frequency U-Net’s multi-scale feature extraction ensures the integrity of the particle core region. A custom collaboration module (Supplementary Algorithm S1) generates the final particle coordinates, which are then superimposed on the original image for visualization. Figure 3b demonstrates the superior performance of the CryoSIP method in protein particle picking. As shown in Supplementary Table S2, CryoSIP extracts 49,646 particles with a low false positive rate (see Fig. 2), outperforming CrYOLO (36,821 particles), Topaz (37,808 particles), and CryoSegNet. According to the 3D reconstruction results in Table 1, CryoSIP achieves a resolution of 3.81 Å, improving by over 1.9 Å compared to the current SOTA model. High-precision particle localization (Precision = 91.4%) and high recall (Recall = 93.0%) are shown to contribute significantly to reconstruction quality. High-precision particle localization (Precision = 91.4%) and high recall (Recall = 93.0%) are shown to contribute significantly to reconstruction quality.

Figure 3c illustrates that CryoSIP effectively recovers fine protein structures. The reconstructed surface exhibits highly continuous and well-defined boundaries, whereas methods like CryoSegNet yield blurred maps with local discontinuities caused by missed or erroneous detections. For example, in the Transport protein, CryoSIP extracts particles spanning a broader conformational space, enabling high-resolution (3.81 Å) reconstruction. In contrast, Topaz suffers from over-detection, introducing non-target particles (Precision = 73.2%), while CrYOLO introduces density errors due to localization inaccuracies (Dice = 82.2%). These results indicate that the multi-frequency semantic segmentation U-Net, combined with SAM’s fine-grained instance segmentation, not only increases particle yield but also enables high-fidelity 3D reconstruction through precise spatial localization (Dice = 91.8%).

Additionally, conducting fair blind evaluations on publicly available raw cryo-EM data from EMPIAR, using the final 3D reconstruction quality as a unified evaluation criterion, is essential for assessing the real-world performance and generalization ability of CryoSIP and competing methods. We collected five non-redundant EMPIAR test datasets corresponding to distinct proteins (EMPIAR-12055, EMPIAR-12010, EMPIAR-12028, EMPIAR-12029, and EMPIAR-12023). None of these datasets were used during the training of CryoSIP or CryoSegNet, and they span a broader range of protein types and resolution levels. We further quantified sequence similarity between training and test proteins using MMseqs2 and applied a stringent 25% sequence identity threshold for redundancy control. Under this criterion, no test proteins exhibited high sequence homology to the training set (sequence identity ≤25%), effectively preventing homologous information leakage and ensuring that the evaluation reflects true model generalization.

Based on these five non-redundant EMPIAR datasets, we systematically evaluated the 3D reconstruction performance of CryoSIP in comparison with other methods from three complementary perspectives. We first compared the resolution of 3D density maps reconstructed by different particle-picking models. We evaluated the performance of CryoSIP, CryoSegNet, CryoTransformer, CASSPER, Topaz, crYOLO, DeepPicker, and a template-based approach in 3D density map reconstruction. For all methods, automatically picked particle coordinates were used as input, followed by an identical reconstruction pipeline in CryoSPARC to generate the final 3D density maps. As shown in Table 4, CryoSIP consistently achieved the highest reconstruction resolution across all datasets. We next assessed the consistency between the reconstructed 3D density maps and their corresponding reference PDB structures. For each reconstructed density map, we computed the Q-score against the corresponding PDB structure to quantitatively assess reconstruction quality. As summarized in Table 5, CryoSIP achieved an average Q-score of 0.45, outperforming all compared methods. We further evaluated the consistency between structures reconstructed by automated modeling and the corresponding reference PDB structures. Automated atomic model building was performed on the reconstructed density maps using Phenix.map_to_model. Reconstruction quality was further assessed by comparing the resulting atomic models with the reference PDB structures. Specifically, identical evaluation metrics were used to compute Cα matching scores between the structures derived from the density maps and the corresponding reference structures. As shown in Table 6, CryoSIP consistently supported the construction of more accurate atomic models.

**Table 4:** Resolution results of 3D density maps reconstructed by various models (Å).

EMPIAR-ID	Template-based	DeepPicker	CrYOLO	Topaz	CASSPER	Cryotransformer	CryoSegNet	CryoSIP
12,055	6.23	8.51	7.74	6.98	6.43	6.50	5.21	4.57
12,010	5.82	7.33	6.39	6.87	6.23	5.76	5.54	5.51
12,028	5.21	6.25	5.27	5.09	5.68	4.87	4.42	4.27
12,029	3.87	4.32	4.10	4.28	3.88	3.85	3.82	3.76
12,023	4.29	6.11	5.98	5.32	4.75	4.12	3.49	3.11
Average	5.08	6.50	5.896	5.71	5.39	5.02	4.50	4.24

**Table 5:** Consistency assessment of reconstructed 3D density maps with corresponding PDB structures (Q-score).

EMPIAR ID	Template-based	Deep Picker	CrYOLO	Topaz	CASSPER	CryoTransformer	CryoSegNet	CryoSIP
10,345	0.35	0.34	0.40	0.42	0.33	0.32	0.41	0.43
10,081	0.38	0.22	0.38	0.36	0.28	0.37	0.38	0.41
10,532	0.41	0.23	0.31	0.36	0.22	0.46	0.51	0.51
10,093	0.21	0.24	0.29	0.21	0.12	0.17	0.31	0.33
12,055	0.25	0.12	0.14	0.19	0.17	0.30	0.42	0.49
12,028	0.34	0.12	0.31	0.32	0.14	0.36	0.43	0.45
12,023	0.41	0.15	0.25	0.31	0.27	0.44	0.52	0.51
Average	0.34	0.20	0.30	0.31	0.22	0.35	0.43	0.45

**Table 6:** Consistency assessment of structures reconstructed by automated modeling software with corresponding PDB structures (cα matching scores).

EMPIAR ID	Template-based	Deep Picker	CrYOLO	Topaz	CASSPER	CryoTransformer	CryoSegNet	CryoSIP
10,345	18.9	22.4	18.0	21.4	5.4	19.6	23.2	23.8
10,081	23.2	22.4	21.7	21.9	15.7	22.7	26.0	27.1
10,532	17.6	13.2	20.3	17.3	12.7	18.2	16.4	18.0
10,093	18.3	3.4	17.0	13.4	5.4	13.6	12.7	14.1
12,055	19.1	15.0	18.9	15.8	12.4	21.2	21.8	23.2
12,028	18.7	14.2	19.3	17	6.9	21.8	22.2	25.1
12,023	23.0	21.1	23.4	22.4	15.4	24.3	24.2	26.5
Average	19.8	16.0	19.8	18.5	10.6	20.2	20.9	22.5

### Key components contribute to both protein particle picking and high-resolution 3D protein reconstruction (RQ3)

To evaluate whether key component designs contribute to protein particle picking and high-resolution 3D reconstruction, three ablation experiments were conducted. (i) Evaluate the role of the multi-frequency domain U-Net framework. (ii) Assess the contribution of key components within the multi-frequency domain U-Net framework. (iii) Investigate the impact of the collaborative optimization mechanism combining coarse-grained semantic and fine-grained instance segmentation.

### Role of the multi-frequency domain U-Net framework

**Figure 4:**
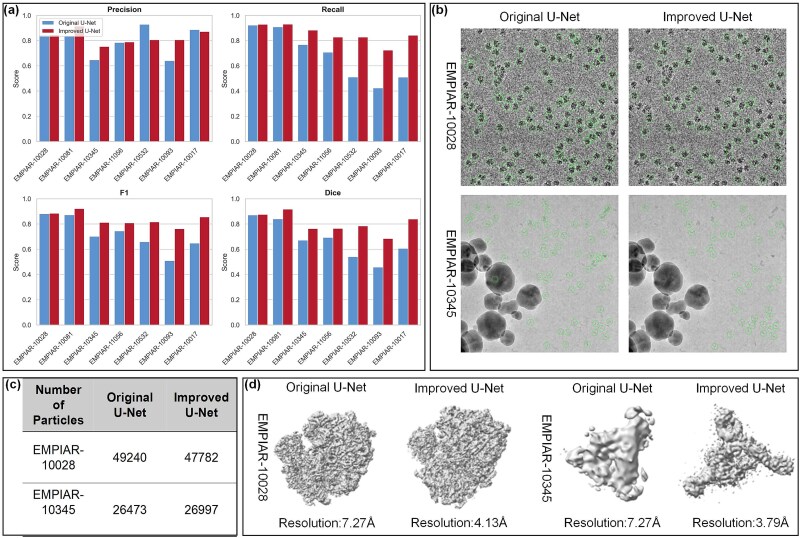
Performance comparison of the original U-Net and the improved multi-frequency domain U-Net in protein particle picking and 3D reconstruction tasks. The figure is divided into four parts: (a) comparison of protein particle picking performance between the original U-Net and the improved multi-frequency domain U-Net on multiple cryo-EM datasets (EMPIAR-10028, −10081, −10345, −11056, −10093, −10017), evaluated by precision, recall, F1-score, and dice coefficient. (b) Visualization of particle picking results for the original U-Net and the improved U-Net on the EMPIAR-10028 and EMPIAR-10345 datasets, with green circle marks indicating the detected particle positions. (c) The number of protein particles detected by the original and improved U-Nets on the EMPIAR-10028 and EMPIAR-10345 datasets. (d) Results of 3D reconstruction of particles extracted using the original and improved U-Nets, respectively.

To validate its effectiveness, we compared the original U-Net with the multi-frequency domain variant on seven cryo-EM protein datasets. Figure 4a shows that the multi-frequency U-Net significantly outperforms the original U-Net across four key metrics for protein particle picking. Specifically, it achieves higher average Precision (82.6% versus 79.5%), Recall (85.1% versus 67.8%), F1-score (83.7% versus 71.6%), and Dice coefficient (80.0% versus 66.8%) (see Supplementary Table S3). The F1-score increased by 12.1% on average, indicating significantly improved adaptability to low-contrast images (Figure 4a). In the Transport dataset (EMPIAR-10081), the multi-frequency U-Net achieved an F1-score of 92.2% (versus 87.2%) and a Dice coefficient of 91.8% (versus 84.1%). In the low signal-to-noise ratio Signaling dataset (EMPIAR-10345), recall improved from 76.8% to 88.2%, and F1-score rose from 70.2% to 81.2%. These improvements result from the multi-frequency feature fusion mechanism, which simultaneously captures global distribution and local details (e.g. particle boundaries), reducing sensitivity to background noise and improving the balance between detection completeness and precision.

The practical value of the enhanced architecture is further demonstrated in downstream 3D reconstruction outcomes. On the EMPIAR-10028 dataset, the number of extracted particles decreased by 1.3% (47 782 versus 49 240) compared to the original U-Net, yet the resolution improved from 4.28 Å to 4.13 Å (see Supplementary Table S3), indicating effective filtering of low-SNR particles via the dynamic mass screening module (Fig. 4b). In contrast, on the EMPIAR-10345 dataset, particle count increased by 2% (26 997 versus 26 473) (Fig. 4c), and resolution improved substantially from 7.27 Å to 3.79 Å (Fig. 4d), confirming the framework’s ability to identify hidden particles. Visual comparisons reveal that the original U-Net caused false positives (e.g. repeated labeling of background regions) due to limited feature representation, whereas the multi-frequency domain U-Net accurately located particles using a cross-band attention mechanism. The resulting reconstructed density map exhibited improved boundary continuity (Fig. 4d), providing higher-fidelity input for structural analysis.

### Role of key components within the multi-frequency domain U-Net framework

To further evaluate the effectiveness of each module in the proposed method, we conducted systematic ablation experiments on the validation set, focusing on two aspects: the data denoising preprocessing module and the internal model architecture. By progressively removing and replacing key components, we assessed the individual contribution of each module to the overall performance.

#### Ablation analysis of data denoising preprocessing

To assess the contribution of the unsupervised denoising module to particle detection, we conducted five comparative experiments, applying different denoising strategies exclusively during data preprocessing, with all other conditions held constant. The five strategies included: no denoising (A1), traditional denoising only (A2), unsupervised denoising only (A3), traditional followed by unsupervised denoising (A4), and unsupervised followed by traditional denoising (A5). Performance was evaluated using precision, recall, F1-score, and Dice coefficient. As shown in Table 7, both traditional denoising (Recall = 84.2%, Dice = 75.1%) and unsupervised denoising (Precision = 62.1%, F1-score = 67.1%) outperformed the undenoised baseline (Precision = 54.5%, Recall = 72.6%, F1-score = 61.8%, Dice = 60.4%), confirming the effectiveness of each method. Notably, combining the two methods further improved performance. The ‘unsupervised followed by traditional’ strategy (Precision = 76.4%, Recall = 91.3%, F1-score = 81.8%, Dice = 76.3%) achieved the best results across all metrics, significantly outperforming all other combinations. These findings highlight both the standalone advantages of unsupervised denoising and its synergistic benefits when combined with traditional methods. In particular, sequential optimization (unsupervised followed by traditional) enhances segmentation precision and particle localization in cryo-EM images.

**Table 7:** Ablation analysis of data denoising preprocessing (%). The five strategies included: no denoising (A1), traditional denoising only (A2), unsupervised denoising only (A3), traditional followed by unsupervised denoising (A4), and unsupervised followed by traditional denoising (A5).

Conditions	Traditional	Unsupervised	Precision	Recall	F1-Score	Dice
A1	X	x	54.5	72.6	61.8	60.4
A2	√	x	69.1	84.2	75.9	75.1
A3	x	√	62.1	73.2	67.1	65.1
A4	√	√	71.3	88.2	78.8	75.3
A5	√	√	76.4	91.3	81.8	76.3

#### Ablation analysis of key components in the model

To assess the contribution of each key module, we performed systematic ablation experiments on the backbone network (see Table 8), evaluating the effects of PVTv2’s pre-trained weights (PW), deep supervision (DS), channel attention (ECA), wavelet convolution (WTConv), and context aggregation (CA). Results show that the baseline model (B1), lacking enhancements, performs poorly (F1 = 58.8%, Dice = 56.4%). With the addition of pre-trained weights (B2), performance improves significantly (F1 = 72.6%, Dice = 70.4%), indicating enhanced feature extraction and faster convergence. Adding deep supervision (B3) further enhances multi-scale feature learning (F1 = 73.7%), while the ECA module (B4) substantially improves channel-wise attention modeling (Precision = 70.2%, F1 = 76.3%). Incorporating WTConv (B5) into the decoder raises the Dice score to 0.750, demonstrating its effectiveness in fine-grained texture enhancement. Removing pre-trained weights while retaining other modules (B6) still leads to a performance drop (Dice = 69.4%), underscoring the critical role of pre-training. Finally, integrating all modules (B7) yields the best performance across all metrics (Precision = 76.4%, Recall = 91.3%, F1 = 81.8%, Dice = 76.3%), demonstrating the cumulative benefit and strong synergy among components. Notably, pre-training and WTConv are essential for effective feature representation and boundary detail restoration, providing critical support for the final architectural design.

**Table 8:** Role of key components within the multi-frequency domain U-Net framework. The modules considered are pre-trained weights (PW), deep supervision (DS), Efficient Channel Attention (ECA), wavelet convolution (WTConv), and context aggregation (CA). Seven configurations are denoted B1–B7: B1 (baseline), B2 (B1 + pre-trained weights), B3 (B2 + deep supervision), B4 (B3 + ECA module), B5 (B4 + WTConv), B6 (all modules enabled except pre-trained weights), and B7 (all modules).

Conditions	PW	DS	ECA	WTConv	CA	Precision	Recall	F1-Score	Dice	Params (M)	Flops (G)	Inference time (ms)
B1	x	x	x	x	x	51.2	69.3	58.8	56.4	29.55	119.87	27.27
B2	√	x	x	x	x	66.0	80.6	72.6	70.4	29.55	119.87	27.27
B3	√	√	x	x	x	67.6	81.2	73.7	71.3	29.55	119.87	31.42
B4	√	√	√	x	x	70.2	83.6	76.3	73.5	29.94	121.14	35.85
B5	√	√	√	√	x	72.3	85.9	78.5	75.0	28.98	113.62	35.60
B6	x	√	√	√	√	65.5	80.2	72.1	69.4	29.02	115.91	36.56

B7	√	√	√	√	√	76.4	91.3	81.8	76.3	29.02	115.91	36.56

Additionally, Table 8 reports per-configuration parameter counts (M), floating-point operations (FLOPs, G; input size 1024 × 1024), and inference time. Across all ablations, parameters span 28.98–29.94 M (≤3% variation), FLOPs 113.62–121.14 G (within ±5%), and inference time is similarly stable. These data indicate comparable computational complexity across settings; thus, the observed performance gains are driven primarily by modular design rather than model size.

#### Role of the semantic-instance collaborative optimization mechanism

To further evaluate the importance of the collaborative optimization mechanism combining coarse-grained semantic segmentation and fine-grained instance boundary correction, two methods were designed to generate .star files. The first method did not use the collaborative optimization mechanism and instead applied SAM to correct the protein particle boundaries based on the semantic segmentation results from the multi-frequency domain U-Net. The second method utilized the collaborative mechanism of coarse-grained semantic segmentation and fine-grained instance boundary correction. First, a coarse-grained mask was generated using multi-frequency domain U-Net to capture the global distribution of particles. Then, the SAM model was introduced to optimize the fine-grained boundary using the coarse-grained mask. Finally, the results of coarse-grained semantic segmentation and fine-grained instance boundary correction were jointly optimized to enhance the accuracy and consistency of the final target area.

**Figure 5:**
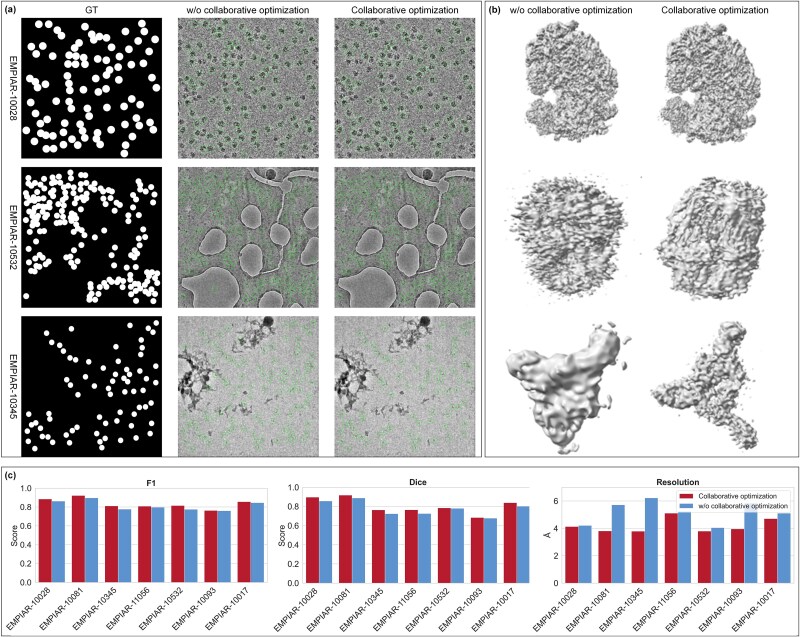
Performance comparison of the model with and without the collaborative optimization mechanism in protein particle segmentation and 3D reconstruction tasks. The results are divided into three main parts: (a) visualization of protein particle picking with and without the collaborative optimization mechanism on the EMPIAR-10028, −10532, and −10345 datasets. The first column shows the ground truth (GT), while the second and third columns display the results of the model with and without the collaborative optimization mechanism. (b) Density maps of protein 3D reconstruction for the EMPIAR-10028, −10532, and −10345 datasets, with and without the collaborative optimization mechanism. (c) Resolution results for protein particle picking and 3D reconstruction. The first, second, and third bar graphs represent the F1 scores, dice coefficients, and resolutions of the models with and without the collaborative optimization mechanism on multiple cryo-EM datasets (EMPIAR-10028, −10081, −10345, −11056, −10093, −10017), respectively.

In both settings, the training process remained consistent, with only the post-processing screening method modified. Evaluation was performed on multiple independent protein datasets, selecting EMPIAR-10028, EMPIAR-10345, and EMPIAR-10532 for visual comparison. The evaluation metrics include Precision, Recall, F1-score, and Dice coefficient. The quantitative evaluation results are shown in Fig. 5c, and the visual results are presented in Fig. 5a and b. As shown in Fig. 5c, the semantic-instance co-optimization mechanism outperforms the original method across all four metrics, particularly in recall and Dice coefficient. This indicates that co-optimization of coarse-grained semantic segmentation and fine-grained instance segmentation effectively eliminates false positives while retaining more true particles, improving both accuracy and completeness. Visual comparison in Fig. 5a clearly shows that, since SAM lacks prior protein information, the absence of the semantic-instance collaborative optimization mechanism results in more false positive particles being detected, including contamination or background areas mistakenly identified as protein particles. With the collaborative optimization mechanism, particle detection becomes more accurate, with better distribution, and a reduction in both missed and false detections. In summary, this ablation experiment demonstrates that the introduction of U-Net and SAM for post-processing collaborative optimization significantly enhances final particle detection performance, which is crucial for subsequent reconstruction tasks (see Fig. 5b and Supplementary Table S4).

Additionally, we evaluated three two-stage variants on seven EMPIAR datasets (IDs: 10028, 10081, 10345, 11056, 10532, 10093, 10017) to quantify the performance gains attributable to the semantic-instance collaborative post-processing. C1 reproduces CryoSegNet: U-Net for semantic segmentation followed by Segment Anything Model (SAM) post-processing to obtain particle masks. C2 keeps the same pipeline as C1 but replaces U-Net with our multi-frequency U-Net. C3 is CryoSIP: our multi-frequency U-Net for segmentation, with SAM-derived masks fused with the U-Net output via semantic-instance consistency to produce the final particles. Detailed results are reported in Supplementary Table S7.

It can be observed that average metrics increase monotonically from C1 to C2 to C3, with the largest gains attributable to collaborative post-processing: relative to C1, C2 improves F1 by 3.0 percentage points (pp), recall by 4.0 pp, and the Dice coefficient by 5.6 pp; building on this, C3 adds a further +4.7 pp F1, +6.4 pp recall, +2.2 pp precision, and + 3.0 pp Dice over C2. Directly comparing C3 with C1, recall rises from 0.747 to 0.851 (+10.4 pp), F1 from 0.761 to 0.838 (+7.7 pp), Dice from 0.719 to 0.804 (+8.6 pp), and precision from 0.792 to 0.826 (+3.4 pp). Across all seven datasets, C3 surpasses C1 in precision and Dice and outperforms C2 in recall and F1.

Gains are especially pronounced for low-SNR or complex-background datasets—EMPIAR-10017, −10093, −10532, and −11056—where recall improves by 22.6, 20.9, 19.9, and 14.0 pp over C1, respectively, without a systematic drop in precision. Mechanistically, we first expand the proposal set by setting the Segment Anything Model (SAM) intersection-over-union (IoU) threshold to ≥0.8; we then enforce semantic consistency between U-Net and SAM (U-Net IoU > 0.3) and apply an adaptive size filter based on the standard deviation of candidate-box diagonal lengths to amplify weak signals while suppressing semantically inconsistent false positives. In sum, the multi-frequency U-Net provides a stronger semantic backbone, whereas the collaborative post-processing is the primary driver of the observed gains. Rather than adopting CryoSegNet’s post-processing, C3 couples a general instance-segmentation model (SAM) with a domain-specific semantic model (U-Net), using semantic priors to constrain geometric proposals and achieving both high recall and high precision under low SNR and complex backgrounds.

#### Reconstruction density and local quality evaluation for EMPIAR-10017 and EMPIAR-10093

**Figure 6:**
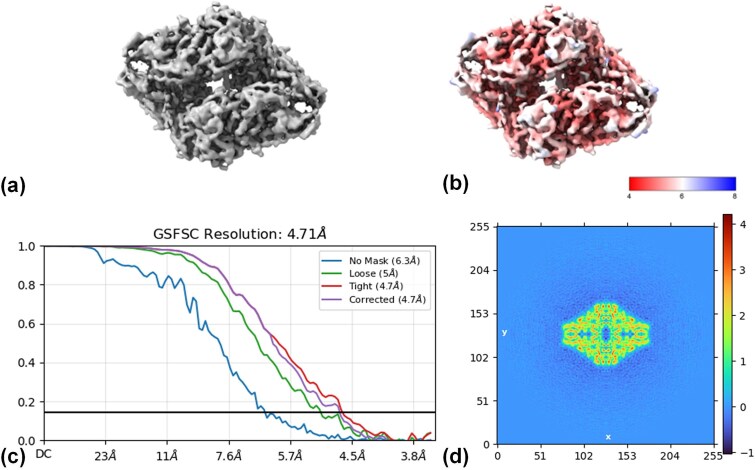
3D reconstruction and resolution analysis of CryoSIP on the EMPIAR-10017 dataset. The analysis is divided into four parts: (a) 3D density map generated by CryoSIP from the EMPIAR-10017 dataset through protein 3D reconstruction. (b) Local resolution distribution of the 3D reconstruction, with red areas indicating high resolution (~4 Å) and blue areas indicating lower resolution (~8 Å). (c) GSFSC resolution curves under various mask conditions, including no mask, loose mask, compact mask, and correction mask. The results show that under the compact mask, the final average resolution reaches 4.71 Å. (d) 2D Fourier space slices, used to evaluate the reconstructed spectral information. High-frequency signals at the image center are clearly visible, and low-frequency signals at the edges are well preserved, indicating the integrity of frequency information during reconstruction.

To further validate the effectiveness of the proposed semantic–instance collaborative particle picking framework for downstream high-resolution reconstruction, we performed 3D reconstructions on two representative datasets: EMPIAR-10017 (***β***-galactosidase) and EMPIAR-10093 (TRPV1 ion channel complex). Specifically, picked particle results were integrated into .star files for 3D reconstruction using the CryoSPARC platform, followed by multi-angle visualization and quantitative evaluation (Fig. 6). Results are presented as 3D density maps, local resolution distributions, GSFSC curves, 2D classification averages, and Fourier density maps. The detailed reconstruction workflow is shown in Supplementary Fig. S3.

For EMPIAR-10017, the 3D reconstruction (Fig. 6a) reveals a highly symmetrical cylindrical protein architecture with well-defined density boundaries, consistent with the known reference structure. The Fourier density map (Fig. 6b) indicates higher resolution in the core region and slightly lower resolution in peripheral regions, reflecting local variations in molecular alignment and averaging accuracy during reconstruction. GSFSC analysis (Fig. 6c) demonstrates that under different masking conditions, the final average resolution reaches 4.71 Å. The real-space slice (Fig. 6d) shows uniform and symmetric cross-sectional density with no apparent reconstruction artifacts, confirming the accuracy and reliability of the 3D reconstruction.

**Figure 7:**
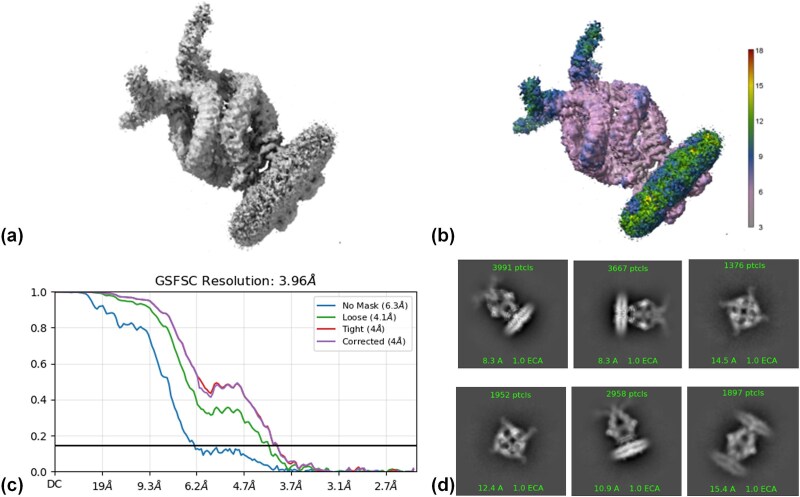
3D reconstruction and resolution analysis of CryoSIP on the EMPIAR-10093dataset. The analysis is divided into four parts: (a) 3D density map generated by CryoSIP from the EMPIAR-10093 dataset through protein 3D reconstruction. (b) Local resolution distribution of the 3D reconstruction, with red areas indicating high resolution (~3 Å) and blue areas indicating lower resolution (~18 Å). (c) GSFSC resolution curves under various mask conditions, including no mask, loose mask, tight mask, and correction mask. The results indicate that under the tight mask, the final average resolution reaches 3.96 Å. (d) Results of 2D particles selected by CryoSIP from multiple directions. Images from various viewing angles show good structural consistency and clarity.

EMPIAR-10093 corresponds to the TRPV1 ion channel complex. As shown in Fig. 7a, the 3D density map reveals a helical configuration of the ion channel complex with clear global contours and rich local details, indicating high particle homogeneity. The local resolution map (Fig. 7b) shows that the core channel region has significantly higher resolution than surrounding flexible areas, consistent with the structural stability of the complex’s functional domain. The final average resolution (Fig. 7c) is 4.06 Å, confirming that the semantic–instance collaborative framework effectively extracts high-quality particles with balanced orientation, supporting high-resolution reconstruction. 2D classification results (Fig. 7d) show consistent and clear average images across multiple viewing angles, further validating the representativeness and stability of particles extracted by the proposed model.

Overall, results from both datasets demonstrate that the CryoSIP model performs robustly not only on ideal datasets but also in real-world cryo-EM scenarios, consistently producing high-quality particles and reconstructions with strong generalization and practical applicability.

## Discussion

The protein particle picking framework proposed in this study significantly enhances picking accuracy and 3D reconstruction quality in cryo-EM images through deep integration of multi-frequency domain segmentation and a semantic–instance collaborative mechanism. The core contributions are manifested in two key aspects. First, the multi-frequency domain U-NET segmentation framework, built on the PVTv2 backbone, enhances robustness against weak boundaries and strong background noise through multi-scale context aggregation, ECA-based channel attention, and improved WTConv. Experiments show that the framework achieves an average F1-score of 83.7% across seven independent test sets—12.1% higher than traditional U-Net. Notably, on low-contrast datasets (e.g. EMPIAR-10345), recall reaches 88.2%, validating its strong generalization. Second, the collaborative mechanism integrating coarse-grained semantic segmentation (multi-frequency domain U-Net) and fine-grained instance segmentation (SAM) addresses the issue of instance adhesion in low SNR images through mask interaction and boundary refinement. For example, the average Dice coefficient increases to 80%, enabling a resolution breakthrough in 3D reconstruction (e.g. EMPIAR-10345 improves from 7.27 Å to 3.79 Å). Additionally, the unsupervised Zero-Shot Noise2Noise (ZS-N2N) denoising strategy, combined with Gaussian filtering, enables structure-preserving preprocessing that suppresses background noise while retaining particle details (e.g. resolution of EMPIAR-10028 improves to 4.13 Å). These innovations offer a high-precision, automated solution for high-throughput cryo-EM analysis.

Despite its strong performance, the CryoSIP framework has several limitations. First, limited sensitivity to small particles (<5 nm) or extremely low-SNR regions may result in missed detections. For instance, recall on EMPIAR-10093 is 72.4%. This is attributed to a training bias toward high-signal particles. Sensitivity to small targets could be improved via multimodal feature learning (e.g. incorporating frequency-domain or gradient-based information). Second, the computational cost of the SAM module reduces inference efficiency, increasing per-image processing time by ~30%. Future work may optimize speed via lightweight hint models or knowledge distillation techniques. Finally, the current framework lacks modeling of particle orientation and 2D class consistency, potentially affecting downstream classification and alignment accuracy. Introducing geometric constraints or self-supervised representation learning may enhance compatibility with downstream tasks.

To address these challenges, future work will focus on three key directions. First, develop an adaptive multimodal architecture that integrates frequency-domain analysis with physical priors to improve robustness in low-SNR particle detection. Second, design a lightweight instance segmentation module (e.g. a SAM variant optimized for edge computing) to balance speed and accuracy. Third, expand modeling of particle attributes (e.g. orientation, morphology) and establish an end-to-end pipeline integrating detection, classification, and reconstruction. The success of this method highlights the potential of deep learning for cryo-EM automation. Its open-source implementation is expected to accelerate structural biology toward higher throughput and resolution, particularly for complex systems such as dynamic assemblies and membrane proteins.

## Materials and Methods

This study proposes an accurate protein particle picking framework tailored for cryo-EM images. To address the core challenges of low contrast, high noise, and blurred boundaries, a comprehensive pipeline is developed, encompassing data preprocessing, mask segmentation refinement, and coordinate screening. In the preprocessing stage, the unsupervised ZS-N2N [27] denoising method enhances the signal-to-noise ratio and local contrast, thereby providing high-quality feature inputs for downstream tasks. In the segmentation stage, a multi-frequency domain U-Net is constructed using PVTv2 [28] as the encoder, which models spatial context via a local–global attention mechanism. The decoder integrates wavelet transforms and residual connections to enhance multi-scale feature reconstruction and boundary restoration. In the optimization stage, we refine the U-Net–SAM collaborative optimization proposed in CryoSegNet [25] by introducing an additional interaction between U-Net and SAM to improve final instance masks and reduce false positives. Spatial overlap and morphological features are jointly optimized to eliminate false positives and artifacts, resulting in a high-confidence particle coordinate set. This pipeline forms a technical loop from noise suppression to precise localization through the collaborative optimization of semantic segmentation (global feature modeling) and instance segmentation (local boundary refinement). The resulting high-confidence coordinate set is exported as a .star file, conforming to standard input requirements for subsequent 3D reconstruction.

### Data preprocessing and augmentation

To address inherent limitations of cryo-EM images—namely, low signal-to-noise ratio, poor contrast, and blurred boundaries—this study develops a multi-stage collaborative optimization pipeline to enhance particle picking quality. The full pipeline is illustrated in Supplementary Fig. S1. First, ZS-N2N is applied for self-supervised denoising, leveraging internal image redundancy to suppress background noise while preserving fine protein structures. Next, a hierarchical denoising chain is applied, including Gaussian filtering (to smooth local noise), FastNLMeans (to preserve edge textures), and Wiener filtering (to suppress high-frequency artifacts), thereby progressively enhancing the signal-to-noise ratio. Subsequently, contrast-limited adaptive histogram equalization (CLAHE) is applied to enhance local contrast. Guided filtering, using the CLAHE output as a reference, fuses the denoised and contrast-enhanced images to sharpen particle boundaries while suppressing background noise. Finally, the pipeline outputs high-fidelity images that preserve structural integrity and feature clarity, serving as input for the downstream segmentation network. This preprocessing strategy enhances image segmentability while avoiding over-smoothing through multimodal filtering and adaptive enhancement techniques.

### Multi-frequency domain U-Net framework

An efficient multi-frequency domain U-Net framework is proposed to achieve accurate semantic segmentation of protein particles in cryo-EM images. The encoder is based on PVTv2. A novel Global–Local Efficient Channel Attention (ECA) mechanism is incorporated to extract both global and local semantic context. The ContextAggregation module integrates multi-scale contextual features to suppress false positives. The decoder incorporates wavelet transforms and residual connections to efficiently aggregate features across scales and frequency domains. The full architecture is illustrated in Supplementary Fig. S4.

#### PVTv2 encoder

The encoder is based on PVTv2 and captures multi-scale global and local contextual information from high-resolution images through hierarchical convolution and attention mechanisms. Its pyramid-based feature extraction is well-suited to the low contrast and high noise in cryo-EM images, offering a robust and discriminative feature foundation for downstream segmentation (see Supplementary Fig. S3 for architecture details).

#### Global–local efficient channel attention

A skip connection module, Global–Local Efficient Channel Attention (Global–Local ECA), is proposed to enhance contextual modeling via dual-path feature fusion. The local path employs depthwise separable convolutions to capture fine-grained textures. The global path captures long-range dependencies using a channel attention mechanism. These two paths jointly suppress background noise and preserve local details, significantly improving segmentation accuracy in low signal-to-noise settings (see Supplementary Fig. S4d for module structure).

#### Aggregation of contexts at different scales

A multi-scale feature aggregation module, ContextAggregation, is introduced to suppress false positives. Multi-scale local features are extracted using dilated convolutions with dilation rates of 1, 3, and 5, while global context is obtained through adaptive global average pooling (GAP). Following feature concatenation, 1 × 1 convolution refines the representations to enhance robustness. This module reduces false detections and improves consistency of the target regions via a scale-adaptive mechanism (see Supplementary Fig. S4e for implementation details).

#### Multi-frequency domain decoder

The decoder integrates wavelet transforms and residual connections, enabling multi-scale detail reconstruction via dual-path fusion in the frequency and spatial domains. The wavelet transform separates high- (boundary) and low-frequency (main) components, while residual connections maintain gradient flow stability. A deep supervision mechanism adds auxiliary loss functions at each decoder layer to facilitate early optimization and hierarchical consistency. Finally, the decoder outputs a high-fidelity segmentation mask (see Supplementary Fig. S4c for architecture details).

### Semantic-instance collaborative protein particle picking

This study introduces a semantic-guided instance adaptation mechanism that significantly enhances the accuracy and reliability of protein particle segmentation in cryo-EM images by integrating coarse-grained semantic segmentation (multi-frequency U-Net) with fine-grained boundary refinement (SAM). Specifically, the SAM is employed to collaboratively refine the preliminary segmentation generated by the multi-frequency U-Net. First, SAM’s general instance segmentation capability is utilized to refine particle mask boundaries, while global semantic context and local instance details are fused using an adaptive weighting strategy (see Supplementary Algorithm S1). Second, candidate instances are filtered based on spatial overlap and morphological consistency (e.g. width, height, area thresholds) to eliminate false positives, such as background noise and non-target artifacts. Finally, center coordinates and radius values of high-confidence particles are extracted and saved in standard .star format to support downstream 3D reconstruction. This collaborative optimization mechanism leverages global semantic features to guide local boundary refinement and uses local feedback to correct global segmentation errors. On datasets such as EMPIAR-10081, this approach improved the F1-score by 9.8% and reduced the false positive rate by 21.3% (see Supplementary Table S1), demonstrating its technical advantages in complex biological samples.

#### Semantic-instance collaborative optimization mechanism

The SAM is a general-purpose segmentation framework pretrained on large-scale visual data. It exhibits zero-shot transfer capabilities and can adaptively segment target objects in complex environments. In this study, SAM’s automatic mask generator was applied to refine the boundaries of preliminary particle masks generated by the multi-frequency U-Net. Uniformly distributed prompt points are automatically supplied to SAM, which then generates high-fidelity candidate masks using pretrained weights (Fig. 1c), significantly improving boundary accuracy in low-contrast regions (e.g. a 42% reduction in boundary error on EMPIAR-10345). Compared to traditional thresholding or morphological operations, SAM leverages self-attention to model global context, effectively addressing particle adhesion and boundary blur in cryo-EM images, thereby enhancing the geometric consistency of segmentation outcomes.

The details of semantic-instance collaborative optimization mechanism are presented in Supplementary Algorithm S1. First, all SAM-generated instances are filtered by prediction confidence; only high-confidence candidates with IoU > 0.80 are retained to exclude background and ambiguous regions. Next, the retained candidates are normalized in size, and morphological constraints based on dominant particle dimensions are applied to exclude abnormally sized noise instances. Finally, instance masks are cross-verified with the U-Net semantic mask; only those with IoU > 0.30 are retained, ensuring both semantic consistency and accurate boundary localization. The final particle coordinates and radii are recorded in a structured **.star** file compatible with reconstruction tools such as CryoSPARC and RELION, enabling direct integration into the downstream single-particle analysis (SPA) pipeline (see Supplementary Fig. S5). On EMPIAR-10081, this mechanism reduced the false positive rate by 19.6% and improved recall by 8.3% (see Supplementary Table S4), validating its collaboration effect.

### Loss function and deep supervision

This study enhances protein particle picking performance through a combination of multi-loss fusion and deep supervision strategies. The loss function combines three components: (i) Binary Cross Entropy (BCE) measures pixel-wise discrepancies for binary classification tasks; (ii) Dice loss assesses the overlap between predicted and true masks, improving target completeness under class imbalance; and (iii) Focal loss down-weights easy negatives and emphasizes hard samples, increasing sensitivity to blurred edges and small particles. The specific loss formulations are defined as follows:


(1) \begin{equation*} {\mathcal{L}}_{BCE}=-\frac{1}{N}\sum_{i=1}^N{y}_i\mathit{\log}\left({p}_i\right)+\left(1-{y}_i\right)\mathit{\log}\left(1-{p}_i\right)\kern-2pt, \end{equation*}

(2) \begin{equation*} {\mathcal{L}}_{\mathrm{Dice}}=1-\frac{2\sum_{\mathrm{i}=1}^{\mathrm{N}}{\mathrm{p}}_{\mathrm{i}}{\mathrm{y}}_{\mathrm{i}}}{\sum_{\mathrm{i}=1}^{\mathrm{N}}{\mathrm{p}}_{\mathrm{i}}+\sum_{\mathrm{i}=1}^{\mathrm{N}}{\mathrm{y}}_{\mathrm{i}}},{\mathcal{L}}_{\mathrm{Focal}}=-\mathrm{\alpha} {\left(1-{\mathrm{p}}_{\mathrm{t}}\right)}^{\mathrm{\gamma}}\log \left({\mathrm{p}}_{\mathrm{t}}\right)\kern-2pt, \end{equation*}

A deep supervision mechanism is designed to improve training efficiency at each decoder stage. Auxiliary BCE and Dice losses are applied at multiple levels to encourage the network to learn fine-grained segmentation features across scales. By comparing predictions with ground truth at each layer (Supplementary Fig. S4b), this approach enforces multi-scale feature consistency and facilitates cross-level gradient propagation. This strategy substantially improves training stability and segmentation accuracy (e.g. a 6.5% F1 increase on EMPIAR-10081), thereby enhancing the model’s generalization in complex scenarios.

The backbone loss of the CryoSIP model comprises Dice Loss, BCE Loss, and Focal Loss. The deep supervision loss includes BCE and Dice losses, and the total loss is calculated as the weighted sum of outputs from all feature layers. The final training objective integrates both backbone and deep supervision losses:


(3) \begin{equation*} {\mathcal{L}}_{total}={\lambda}_1{\mathcal{L}}_{BCE}+{\lambda}_2{\mathcal{L}}_{Dice}+{\lambda}_2{\mathcal{L}}_{Focal}+\sum_{j=1}^3{\mu}_j\cdot \left({\mathcal{L}}_{BCE}^j+{\mathcal{L}}_{Dice}^j\right)\kern-2pt, \end{equation*}

where ${\mathrm{\lambda}}_1$, ${\mathrm{\lambda}}_2$ and ${\mathrm{\lambda}}_3$ represent the weights for Dice, BCE, and Focal losses, respectively, set to 1, 1, and 0.5 by default. The weight for the output loss at the ***j***-th decoder layer is set to 0.4, 0.3, and 0.2, respectively.

**Key Points**

- We introduce CryoSIP, a semantic–instance collaborative framework that improves protein particle picking under low SNR and weak contrast.
- A multi-frequency adaptive U-Net integrates global contextual semantics and multi-scale features to localize particles precisely.
- We refine the U-Net–SAM collaboration by adding an interaction stage to improve instance masks and reduce false positives.

## Supplementary Material

Supplementary_materials(BIB)_bbag138

## Data and materials availability

The code and data are available at: https://github.com/xiachashuanghua/CryoSIP.
