# Supplementary material for: CryoSIP: unleashing protein high-resolution Cryo-EM via semantic-instance collaborative picking
Source: Brief Bioinform. 2026 Apr 1;27(2):bbag138. doi: 10.1093/bib/bbag138 (PMC13043001; doi:10.1093/bib/bbag138)
Supplement: Supplementary_materials(BIB)_bbag138 [file supplementary_materials(bib)_bbag138.pdf]

Supplementary Materials for

CryoSIP: Unleashing Protein High-Resolution Cryo-EM via Semantic-Instance Collaborative Picking

Yu Deng *et al.*

This PDF file includes:

Supplementary Notes 1 to 4
Supplementary Figures S1 to S5
Supplementary Tables S1 to S7

Supplementary Note 1

Semantic-instance collaborative optimization algorithm

This study introduces a semantic-guided instance adaptation mechanism that significantly enhances the accuracy and reliability of protein particle segmentation in cryo-EM images by integrating coarse-grained semantic segmentation (multi-frequency U-Net) with fine-grained boundary refinement (SAM). Specifically, the SAM is employed to collaboratively refine the preliminary segmentation generated by the multi-frequency U-Net. First, SAM's general instance segmentation capability is utilized to refine particle mask boundaries, while global semantic context and local instance details are fused using an adaptive weighting strategy (see Supplementary Algorithm X). Second, candidate instances are filtered based on spatial overlap and morphological consistency (e.g., width, height, area thresholds) to eliminate false positives, such as background noise and non-target artifacts. Finally, center coordinates and radius values of high-confidence particles are extracted and saved in standard .star format to support downstream 3D reconstruction. The detailed Semantic-instance collaborative optimization process is shown in **Algorithm S1.**

Algorithm S1 proceeds in three stages: Step 1 uses the SAM to generate an initial set of protein-particle candidates (seven sub-steps, 1.1–1.7); Step 2 fuses these candidates with the U-Net segmentation to obtain a refined set (three sub-steps, 2.1–2.3); Step 3 exports the final particle coordinates and sizes to a .star file.

Additionally, we analyze CryoSIP's Supplementary Algorithm 1 alongside CryoSegNet's Supplementary Algorithm S1 (post-processing). Although both methods use the Segment Anything Model (SAM) to generate initial instance masks, their post-processing strategies differ fundamentally. CryoSegNet's S1 relies solely on SAM outputs: it retains masks with predicted IoU > 0.94 and applies a fixed tolerance (±10% of the modal bounding-box diameter). This heuristic removes some noise but has key limitations: (i) because SAM is not trained on cryo-EM data, it can propose plausible-looking particle masks in regions where the U-Net assigns low semantic confidence (e.g., blurred background or ice-crystal edges), which should be suppressed; and (ii) SAM may also produce masks in areas not segmented by the U-Net at all (non-protein regions), which IoU and size constraints alone cannot detect. Without domain-semantic guidance, CryoSegNet struggles to distinguish "particle-like but non-protein" structures, leading to elevated false positives or requiring an excessively high IoU threshold that sacrifices weak-signal true particles.

In contrast to CryoSegNet, CryoSIP's central innovation is a U-Net–guided semantic module that enables a synergistic "proposal generation + semantic verification" pipeline. We relax the Segment Anything Model (SAM) proposal filter to IoU > 0.8 to admit more potential true positives and then enforce semantic consistency by computing the IoU between each SAM-derived (circularized) particle proposal and the U-Net segmentation, retaining only candidates with U-Net overlap > 0.3. Because the U-Net is trained on large-scale cryo-EM data, it encodes a protein-specific semantic prior and suppresses non-protein structures (e.g., ice crystals and carbon-film textures) that SAM alone cannot disambiguate, thereby reducing false positives. To improve robustness, CryoSIP replaces CryoSegNet's fixed ±10% size tolerance with an adaptive criterion based on the standard deviation of proposal diagonal lengths, which automatically adjusts the acceptance window to the observed particle-size dispersion across micrographs and imaging conditions. Finally, CryoSIP applies a three-stage cascade—confidence filtering (IoU > 0.8), adaptive size screening, and semantic-consistency verification (U-Net IoU > 0.3)—to mitigate single-step failure while preserving weak-signal particles: proposals with modest SAM

confidence are retained when they exhibit strong agreement with U-Net, avoiding the loss of particles essential for high-resolution reconstruction.

Algorithm S1. Particle picking based on semantic-instance collaborative optimization

Input: output mask set of SAM ($\boldsymbol{M_{SAM}}$), output mask set of U-Net ($\boldsymbol{M_{UNet}}$), the original input image size is $\langle \boldsymbol{o_w}, \boldsymbol{o_h} \rangle$.

Output: A **.star** file containing all the screened particles

---

Process:

Step 1: Initial Screening of Protein Particle Set ($\boldsymbol{P_1}$)

1.1 Extract SAM-predicted masks into set $\boldsymbol{M_{SAM}}$ with **IoU** > 0.8 and remove background particles.

1.2 For each bounding box in $\boldsymbol{M_{SAM}}$, record $\langle \boldsymbol{s_x}, \boldsymbol{s_y}, \boldsymbol{s_w}, \boldsymbol{s_h} \rangle$ and collect them into the set $\boldsymbol{BM} = \{\langle \boldsymbol{s_x}, \boldsymbol{s_y}, \boldsymbol{s_w}, \boldsymbol{s_h} \rangle\}$. $\langle \boldsymbol{s_x}, \boldsymbol{s_y} \rangle$ is bounding box coordinate, $\langle \boldsymbol{s_w}, \boldsymbol{s_h} \rangle$ is size.

1.3 Compute the mode of widths and heights in $\boldsymbol{BM}$ as $\boldsymbol{m_w}$ and $\boldsymbol{m_h}$, respectively.

1.4 Compute the standard deviation of the diagonal lengths of all boxes in $\boldsymbol{BM}$ as $\boldsymbol{std_l}$.

1.5 Rescale the particle's width and height to the original image size and estimate its diameter using the equation: $\boldsymbol{d} = \sqrt{\left\{\left(\boldsymbol{m_w} \cdot \frac{\boldsymbol{o_w}}{\boldsymbol{1024}}\right)\right\}^2 + \left\{\left(\boldsymbol{m_h} \cdot \frac{\boldsymbol{o_h}}{\boldsymbol{1024}}\right)\right\}^2}$.

1.6 Define the screening tolerance threshold ($\boldsymbol{th}$) based on the particle diameter ($\boldsymbol{d}$) and the standard deviation of candidate box diagonals ($\boldsymbol{std_l}$), as described by:

$$\boldsymbol{th} = \boldsymbol{0.0625} * \boldsymbol{d} + \boldsymbol{0.005} * \boldsymbol{std_l}$$

1.7 Retain particles whose width and height satisfy the following conditions as set $\boldsymbol{P_1}$, as defined by: $\boldsymbol{m_w} - \frac{\boldsymbol{th}}{\boldsymbol{3}} < \boldsymbol{s_w} < \boldsymbol{m_w} + \boldsymbol{th}, \boldsymbol{m_h} - \frac{\boldsymbol{th}}{\boldsymbol{3}} < \boldsymbol{s_h} < \boldsymbol{m_h} + \boldsymbol{th}$

Step 2: Refined Screening of Protein Particle Set ($\boldsymbol{P_2}$)

2.1 Compute the **IoU** between the circular mask of the particles in $\boldsymbol{P_1}$ and $\boldsymbol{M_{UNet}}$, retain those with **IoU**> 0.3, and store them in set $\boldsymbol{P_2}$.

2.2 Map each particle in P_2 to its original image coordinates $\langle \boldsymbol{x}, \boldsymbol{y} \rangle$:

$$\boldsymbol{x_{new}} = \left(\frac{s_x + s_w/2}{\boldsymbol{1024}}\right) \cdot \boldsymbol{o_w}, \boldsymbol{y_{new}} = \left(\frac{s_y + s_h/2}{\boldsymbol{1024}}\right) \cdot \boldsymbol{o_h}.$$

2.3 Update each particle in $\boldsymbol{P_2}$ with new coordinates and size $\langle \boldsymbol{x_{new}}, \boldsymbol{y_{new}}, \boldsymbol{d} \rangle$.

Step 3: Write the coordinates and size of all $\boldsymbol{P_2}$ particles to a **.star** file.

Algorithm S1.

Supplementary Note 2

Gold standard and positive-negative sample definitions

CryoPPP establishes its gold standard through a multi-stage, expert-led workflow with structural verification: two cryo-EM experts manually pick particles from ~20 micrographs per EMPIAR dataset; the picks undergo 2D classification, and high-quality classes are selected based on resolution, particle count, and visual plausibility; these classes serve as templates for automated template matching across ~300 micrographs; subsequent manual review adjusts the normalized cross-correlation (NCC) and local-power thresholds to remove false positives (e.g., ice crystals, aggregates, carbon films) while retaining high-confidence particles; this procedure is iterated until a set suitable for high-resolution 3D reconstruction is obtained. Positive samples are particles validated by 2D/3D structure, whereas negatives are typical false-positive regions (e.g., ice contamination, protein aggregates, carbon edges) explicitly flagged during review and saved separately (negative.star) to facilitate junk identification.

Annotation quality was further verified by comparison with the "gold-standard" particles released by EMPIAR depositors (e.g., EMPIAR-1034554 and EMPIAR-1040655), for which 3D density maps reconstructed from CryoPPP picks achieved equal or superior resolution to the originals; inter-annotator agreement between the two experts was also confirmed. Accordingly, we directly adopt CryoPPP's positive/negative labels for training and evaluation; the gold-standard construction is fully disclosed and reproducible in the original literature.

Supplementary Note 3

Experimental Setup

To enhance model generalization, various data augmentation strategies were applied during training, including random rotation, flipping, brightness and contrast adjustments, gamma correction, CLAHE equalization, Gaussian blur, and sharpening, simulating realistic image distortions and noise. Network optimization employed the AdamW optimizer (initial learning rate = 0.001) with a CosineAnnealingWarmRestarts scheduler to dynamically adjust learning rates and mitigate overfitting. The loss function integrates BCE, Dice, and Focal losses, improving sensitivity to small particles by applying class-weighted balancing. A batch size of 7 was used over 200 training epochs. Model performance was evaluated on the validation set after each epoch to guide parameter updates. Additionally, mixed-precision training and the GradScaler mechanism in PyTorch were employed to accelerate convergence and reduce memory usage, ensuring efficient and stable model training.

The model's performance was comprehensively evaluated using four core metrics: precision, recall, F1-score, and Dice coefficient. True positives (TP) denote correctly identified particles, false positives (FP) refer to incorrectly detected particles, and false negatives (FN) indicate missed targets. Precision measures detection reliability, indicating the proportion of predicted particles that are true targets—crucial in applications with low tolerance for false positives. Recall assesses detection completeness, measuring how many true particles are successfully identified, which is especially important for low-SNR or sparse samples. The F1-score combines precision and recall via a harmonic mean, reflecting the model's ability to balance accuracy and completeness. The Dice coefficient quantifies spatial overlap between predicted masks and ground truth annotations, serving as a key indicator of boundary localization accuracy. In cryo-EM tasks, precision emphasizes false positive reduction, recall addresses missed detections, F1-score balances both aspects, and Dice highlights geometric consistency. Together, these four metrics constitute a comprehensive and complementary evaluation framework. The definitions of each metric are provided below:

$$\boldsymbol{Precision} = \frac{\boldsymbol{TP}}{\boldsymbol{TP}+\boldsymbol{FP}}, \boldsymbol{Recall} = \frac{\boldsymbol{TP}}{\boldsymbol{TP}+\boldsymbol{FN}}, \boldsymbol{Dice} = \frac{2\times\boldsymbol{TP}}{2\times\boldsymbol{TP}+\boldsymbol{FP}+\boldsymbol{FN}},$$
$$\boldsymbol{F1-score} = \frac{2\times\boldsymbol{Precision}\times\boldsymbol{Recall}}{\boldsymbol{Precision}+\boldsymbol{Recal}}, \tag{1}$$

Supplementary Note 4
Choices of training and test datasets

All training and test data are drawn from the CryoPPP benchmark, a systematic, expert-curated cryo-EM resource comprising 34 representative protein datasets with micrographs and unified, high-quality annotations. CryoPPP is appropriate because its data quality and consistent labels support reliable, reproducible evaluation, and its breadth of experimental conditions and particle types makes it suitable for benchmarking general particle-picking algorithms.

For training datasets, we selected 22 CryoPPP datasets to balance efficiency and diversity, guided by: (i) diversity — coverage of multiple protein classes (e.g., membrane and signaling proteins), a wide size range (approx. small ~118 px, medium ~313 px, large ~730 px), and varied SNR and background complexity; (ii) representativeness — emphasis on common experimental conditions to learn generalizable priors; and (iii) scalability — matching training scale and heterogeneity to available GPU resources to enable smooth scaling without prohibitive cost.

For testing datasets, we chose seven challenging yet representative datasets to yield discriminative evaluations under constrained resources, adhering to: (i) challenge and scene coverage — datasets include low SNR, particle adhesion, and complex backgrounds to stress robustness; (ii) independence and fairness—test sets are strictly held out from training; and (iii) quality and diversity—despite the smaller scale, annotations are high-quality and scenes diverse, providing a prudent and effective assessment of applicability and robustness.

Additionally, we did not adopt a larger dataset for three reasons: (i) annotation quality and standardization — EMPIAR is extensive, but many entries lack standardized particle labels, whereas CryoPPP provides higher-quality, unified annotations suitable for reliable training and evaluation; (ii) computational constraints — scaling training and testing substantially increases storage and GPU demand, and under current conditions parallel processing within a single research cycle is infeasible; and (iii) experimental strategy—we prioritized representative, high-quality annotated data to rigorously validate the method. Resources permitting and as standardized annotations become more complete, we will extend to larger datasets to more comprehensively assess model generalization.

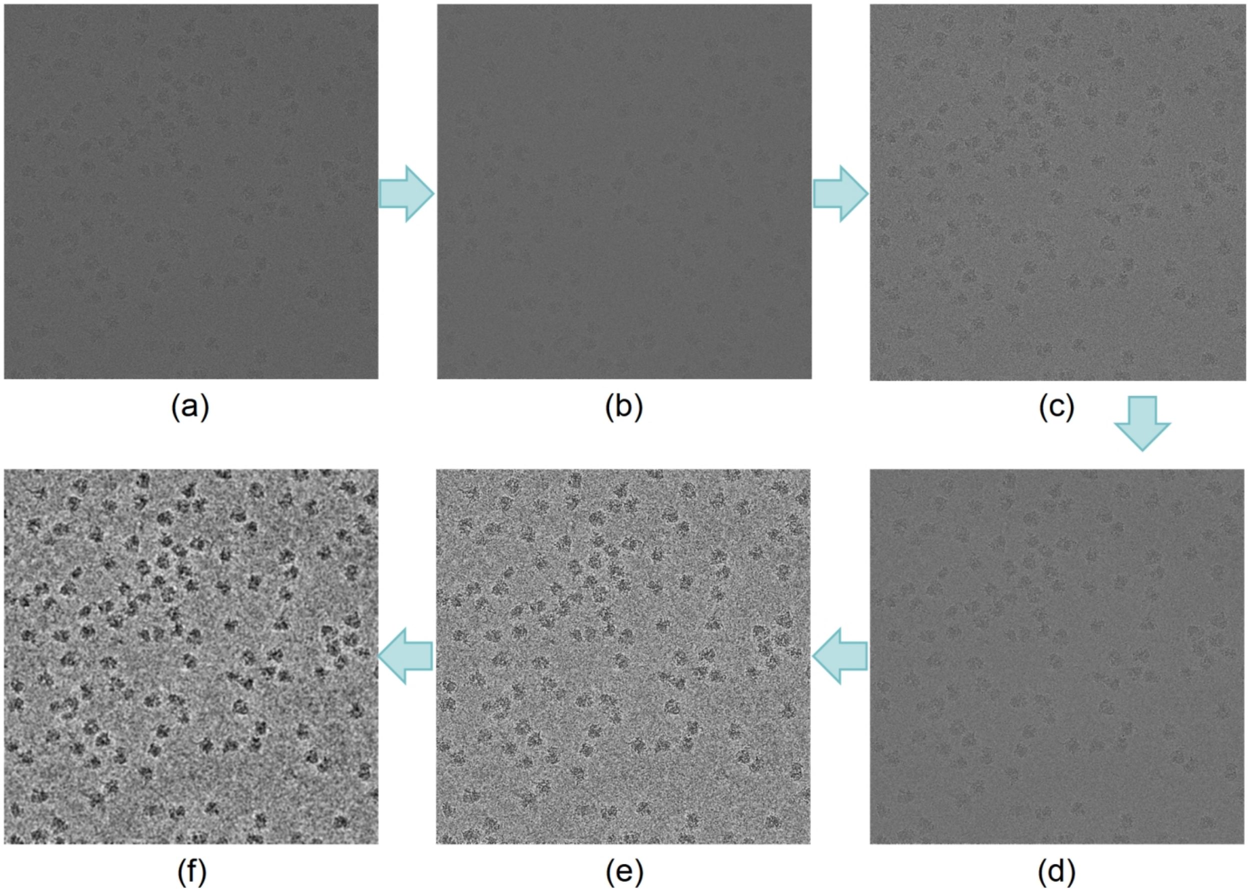

Figure S1.

Image denoising workflow of CryoSIP on the EMPIAR-10093 dataset. (a) Original cryo-EM image with low contrast and a low signal-to-noise ratio. (b) Denoised image using the unsupervised Zero-Shot Noise2Noise (ZS-N2N) framework. (c) Standardized cryo-EM image after normalization. (d) Image further denoised using the FastNLMeans technique. (e) A Wiener filter is applied to further denoise (d), followed by contrast enhancement using CLAHE. (f) Guided filtering is applied to the Wiener-filtered image, using (e) as the guidance. This multi-level, multi-strategy denoising scheme substantially enhances image quality.

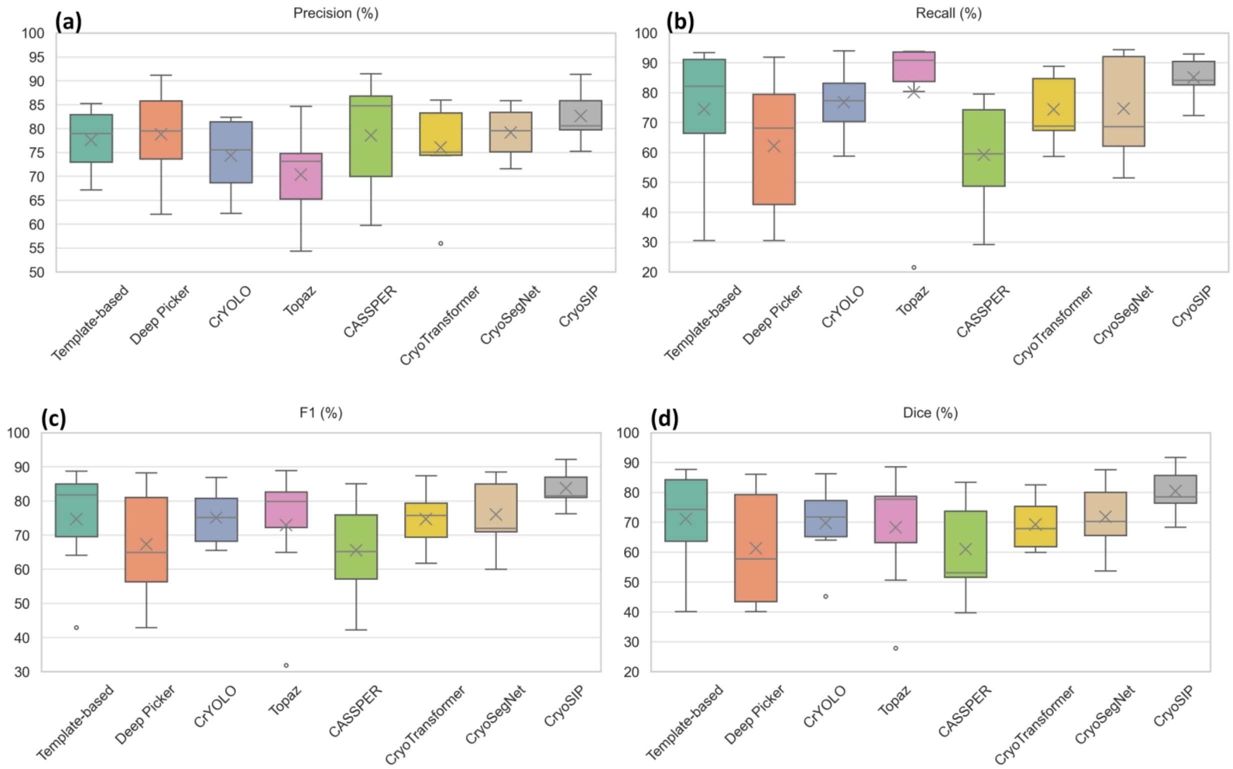

Figure S2.

The box plots of protein particle picking performance across multiple cryo-EM datasets (EMPIAR-10028, -10081, -10345, -11056, -10093, -10017) using various methods, evaluated by **(a)** Precision, **(b)** Recall, **(c)** F1-score, and **(d)** Dice coefficient.

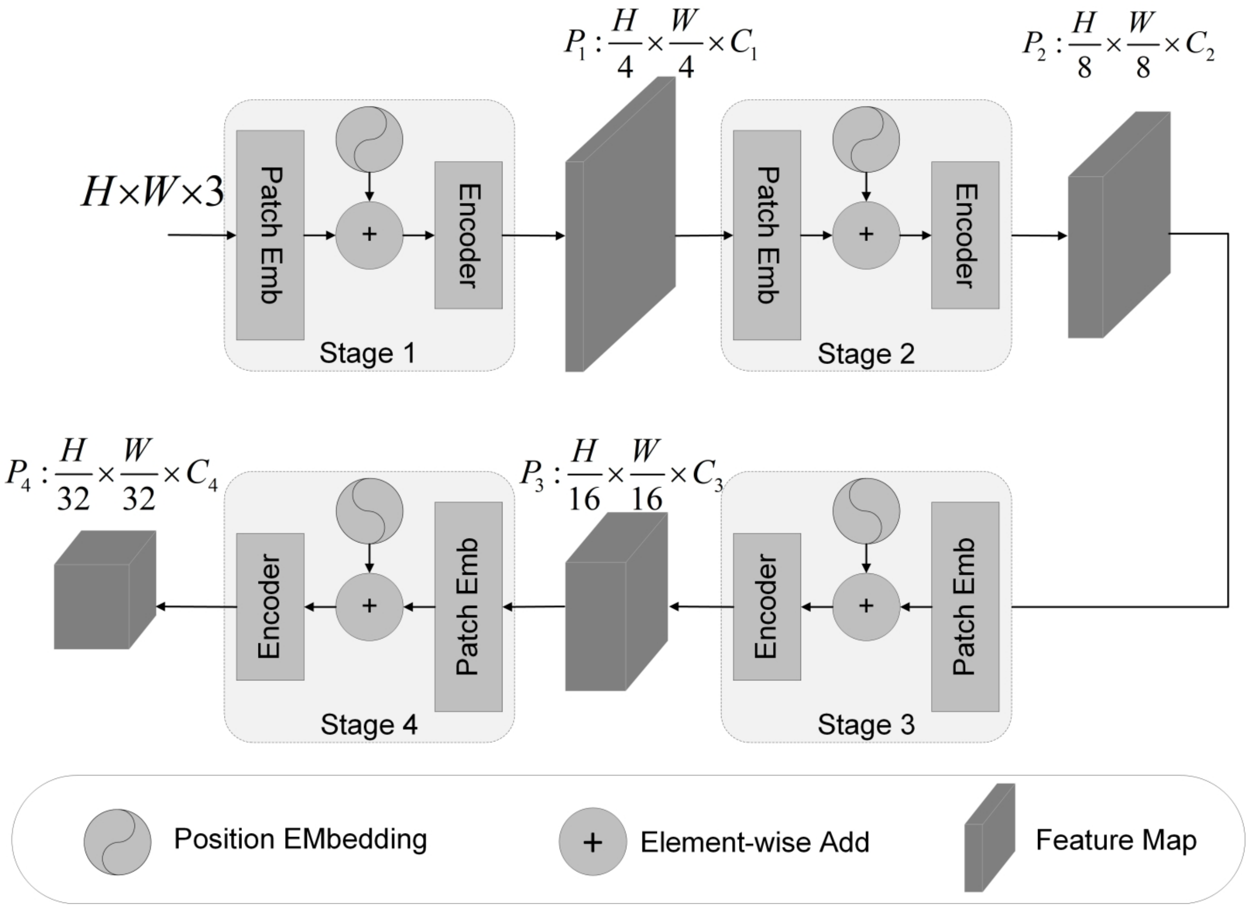

Figure S3.

Leveraging the multi-scale feature extraction architecture of Pyramid Vision Transformer v2 (PVTv2), this approach enables effective multi-scale feature extraction and global context modeling of cryo-electron microscopy images via positional embedding, patch embedding, and efficient encoding, thereby facilitating accurate protein particle segmentation.

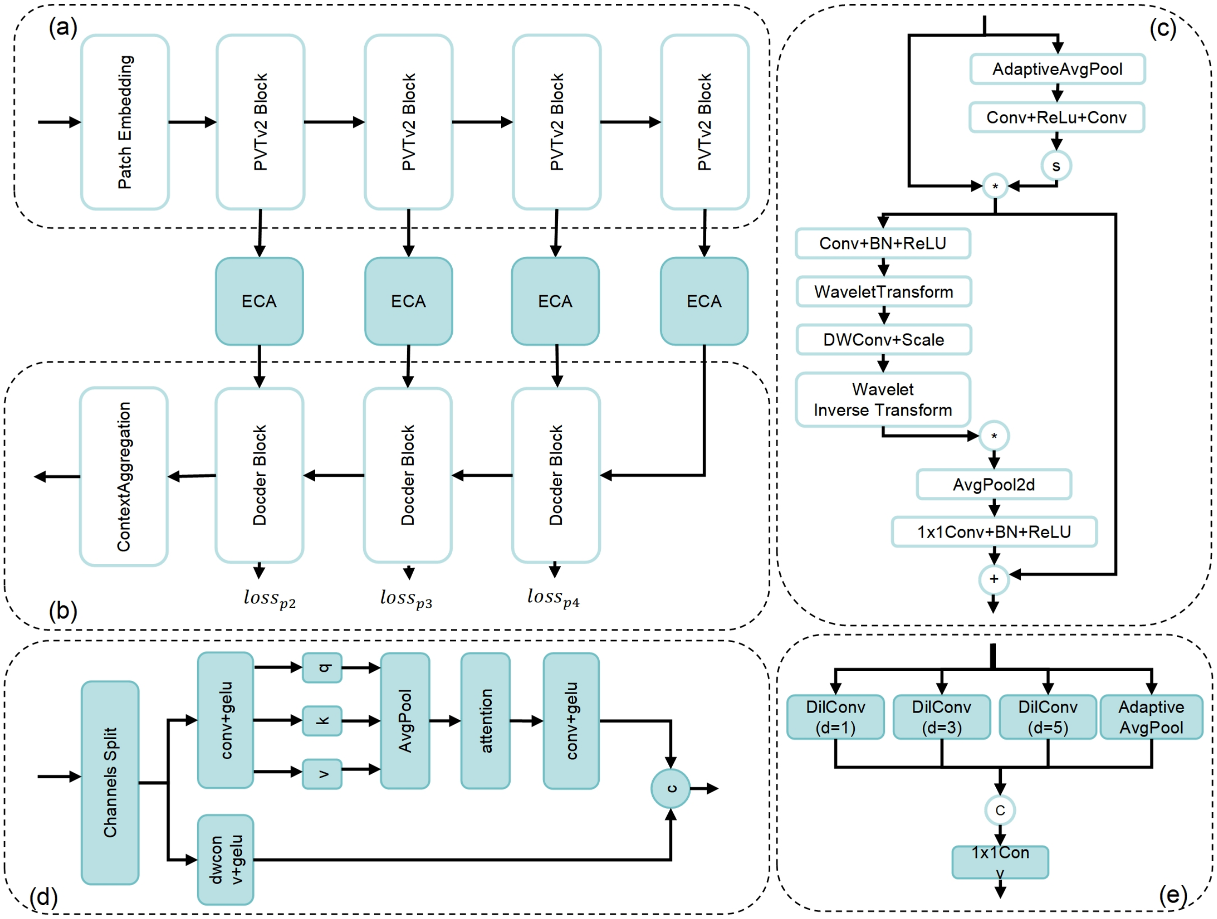

Figure S4.

The multi-frequency U-Net in CryoSIP consists of five key modules (**a** – **e**), collaboratively enabling high-precision segmentation of protein particles in cryo-EM images. **(a)** PVTv2 Encoder: Provides rich semantic features to support the decoding process. **(b)** Decoder Architecture: Incorporates deep supervision to guide multi-scale learning of fine-grained features. **(c)** Decoder Block: Employs a multi-frequency enhancement strategy, applying wavelet transform to decompose the image into different frequency components. These are further refined via depthwise separable convolutions and scale operations to improve edge segmentation accuracy. **(d)** Global-Local ECA Attention: Enhances contextual modeling by fusing global and local features, guiding the model to focus on protein-relevant regions. **(e)** Context Aggregation Module: Combines dilated convolutions (d = 1, 3, 5) and adaptive pooling to capture multi-scale receptive fields and model particle shape and boundaries comprehensively. Overall, CryoSIP enhances segmentation performance through hierarchical feature extraction, multi-frequency enhancement, attention mechanisms, and multi-scale fusion strategies.

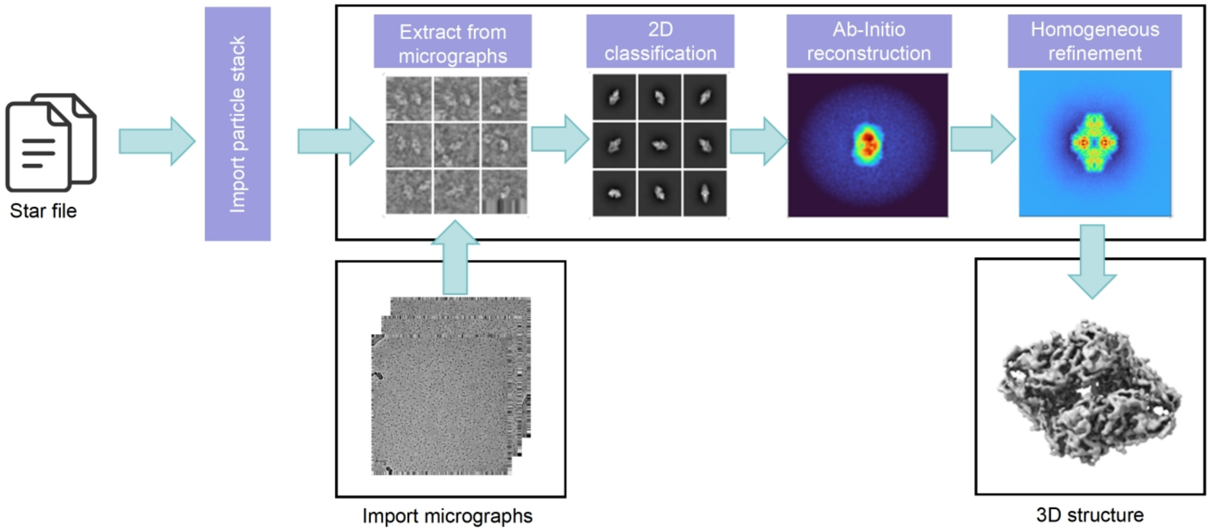

Figure S5.

Using the **.star** file generated by CryoSIP, the full pipeline of protein 3D reconstruction is performed, including particle extraction, classification, initial reconstruction, refinement, and final 3D structure output.

Table S1.

Comparison of protein particle picking performance across multiple cryo-EM datasets (EMPIAR-10028, -10081, -10345, -11056, -10093, -10017) using various methods, evaluated by Precision, Recall, F1-score, and Dice coefficient. This table presents the raw data corresponding to **Figure 2** in the manuscript and summarizes the statistical characteristics of the dataset. M1 – M8 correspond to the Template-based, Deep Picker, CrYOLO, Topaz, CASSPER, CryoTransformer, CryoSegNet, and CryoSIP models, respectively.

Datasets	Type of protein	Images	Particles	Metrics	M1	M2	M3	M4	M5	M6	M7	M8
10028	Ribosome (80S)	300	26391	Precision	84.5	84.8	80.7	69.6	87.7	75.1	83.3	84.6
				Recall	93.5	92	94.1	93.7	59.1	83.2	94.4	92.8
				F1-Score	88.8	88.3	86.9	79.9	70.6	78.9	88.5	88.5
				Dice Score	87.2	86.1	86.3	78.6	66.8	82.6	85.9	87.6
10081	Transport	300	39352	Precision	85.3	86.8	82.2	73.2	91.5	86.0	83.5	91.4
				Recall	90.0	90.0	88.4	87.2	79.6	88.9	92.2	93.0
				F1-Score	87.6	88.3	85.2	79.6	85.1	87.4	87.6	92.2
				Dice Score	87.7	82.2	82.2	75.8	83.4	82.3	87.6	91.8
10345	Signaling	295	15894	Precision	74.3	91.2	64.8	54.4	59.8	74.4	74.6	75.3
				Recall	92.4	50.4	66.5	80.5	71.6	86.4	92.0	88.2
				F1-Score	82.4	64.9	65.6	65.0	65.2	79.9	82.4	81.2
				Dice Score	70.7	57.8	45.2	50.7	53.2	68.4	74.3	76.4
11056	Transport	305	125908	Precision	71.7	71.7	72.6	76.4	76.8	85.3	75.7	78.9
				Recall	30.6	30.6	78.0	90.9	29.2	68.3	68.7	82.7
				F1-Score	42.9	42.9	75.2	83.0	42.3	75.8	72.0	80.8
				Dice Score	40.1	40.1	71.8	77.8	39.8	67.9	66.3	76.5
10532	Viral	300	87933	Precision	79.0	62.1	75.6	73.2	84.8	81.3	79.6	80.6
				Recall	71.6	68.2	77.4	93.9	38.5	66.5	62.8	82.7
				F1-Score	75.1	65.0	76.5	82.3	53.0	73.2	70.2	81.6
				Dice Score	74.3	43.2	72.4	78.8	51.9	61.4	64.9	78.5
10093	Membrane	295	56394	Precision	67.2	75.6	62.3	61.0	63.2	56.0	71.6	80.6
				Recall	61.3	34.9	74.4	21.6	59.6	68.9	51.5	72.4
				F1-Score	64.1	47.8	67.8	31.9	61.3	61.8	60.0	76.3
				Dice Score	56.8	43.7	64.1	27.9	51.3	60.0	53.7	68.4
10017	***β***-Galactosidase	84	49391	Precision	81.4	79.5	82.4	84.7	86.0	74.5	85.9	87.1
				Recall	82.2	69.1	58.8	93.6	77.0	58.7	61.6	84.2
				F1-Score	81.8	73.9	68.6	88.9	81.3	65.7	71.8	85.6
				Dice Score	81.3	76.4	66.3	88.6	80.8	62.3	70.3	83.9
Average				Precision	77.6	78.8	74.4	70.4	78.5	76.1	79.2	82.6
				Recall	74.5	62.2	76.8	80.2	59.2	74.4	74.7	85.1
				F1-Score	74.7	67.3	75.1	72.9	65.5	74.7	76.1	83.7
				Dice Score	71.2	61.4	69.8	68.3	61.0	69.3	71.9	80.0

Table S2.

The number of protein particles picked up by Template-based, Deep Picker, CrYOLO, Topaz, CASSPER, CryoTransformer, CryoSegNet, and CryoSIP models respectively.

EMPIAR ID	Number of Particles							
	Template-based	Deep Picker	CrYOLO	Topaz	CASSPER	CryoTransformer	CryoSegNet	CryoSIP
10028	32183	30242	31699	35514	15637	40488	49240	47782
10081	41569	28209	36821	37808	27299	88632	60256	99229
10345	14353	2470	11369	21343	9876	105739	26473	26997
11056	53190	17124	43599	66651	34860	98193	76106	104532
10532	43662	28711	29434	38372	29290	148345	35779	79049
10093	42986	2360	33183	61698	32383	151545	48134	42913
10017	49770	23462	47704	45511	38460	43735	11965	28173
Average	39673	18940	33401	43842	26829	96668	43993	68828

Table S3.

The CryoSIP model compares the original U-Net with the proposed multi-frequency U-Net framework across multiple cryo-EM datasets. The comparison focuses on protein particle picking (measured by Precision (%), Recall (%), F1-score (%), and Dice coefficient (%)) and 3D reconstruction (evaluated by Resolution (Å)). This table presents the raw data underlying **Figure 4** of the manuscript.

Datasets/metrics		Precision	Recall	F1-score	Dice	particles	Resolution (Å)
Original U-Net	10028	84.4	92.2	88.1	87.1	49240	4.28
	10081	83.7	90.9	87.2	84.1	50514	4.48
	10345	64.7	76.8	70.2	67.1	26422	7.27
	11056	78.5	70.8	74.5	69.3	95601	9.32
	10532	92.9	51.1	65.9	54.1	38525	4.47
	10093	64.1	42.4	51.0	45.8	54846	6.91
	10017	88.7	51.0	64.8	60.7	20810	6.04
	Average	79.5	67.8	71.6	66.8	47994	6.11
Improved U-Net	10028	84.6	92.8	88.5	87.6	47782	4.13
	10081	91.4	93.0	92.2	91.8	99229	3.80
	10345	75.3	88.2	81.2	76.4	26997	3.79
	11056	78.9	82.7	80.9	76.5	104532	5.10
	10532	80.6	82.7	81.6	78.5	79049	3.80
	10093	80.6	72.4	76.3	68.4	42913	3.96
	10017	87.1	84.2	85.6	83.9	28173	4.70
	Average	82.6	85.1	83.7	80.0	68828	4.19

Table S4.

The CryoSIPPerformance comparison of the model **with** and **without (w/o)** the collaborative optimization mechanism in protein particle picking (measured by Precision (%), Recall (%), F1-score (%), and Dice coefficient (%)) and 3D reconstruction (evaluated by Resolution (Å)). This table presents the raw data underlying **Figure 5** of the manuscript.

Datasets/metrics		Precision	Recall	F1-score	Dice	Resolution (Å)
**w/o** collaborative optimization	10028	81.7	91.5	86.3	85.7	4.21
	10081	90.0	89.4	89.7	88.9	5.72
	10345	72.3	84.2	77.8	72.4	6.23
	11056	78.0	81.7	79.8	72.6	5.24
	10532	76.5	78.8	77.6	78.0	4.05
	10093	80.3	72.2	76.0	67.8	5.84
	10017	86.4	82.8	84.6	80.3	5.11
	Average	80.7	82.9	81.7	77.5	5.20
collaborative optimization	10028	84.6	92.8	88.5	87.6	4.13
	10081	91.4	93.0	92.2	91.8	3.80
	10345	75.3	88.2	81.2	76.4	3.79
	11056	78.9	82.7	80.9	76.5	5.10
	10532	80.6	82.7	81.6	78.5	3.80
	10093	80.6	72.4	76.3	68.4	3.96
	10017	87.1	84.2	85.6	83.9	4.70
	Average	82.6	85.1	83.7	80.0	4.19

Table S5.

Overview of the 22 training datasets (* indicates the theoretical weight of the protein). In the experiment, the 22 datasets were integrated into a unified training set.

SN	EMPIAR ID	Type of Protein	Image Size	Total Structure Weight (kDa)	Training Images	Validation Images	Total Images
1	10005	TRPV1 Transport Protein	(3710, 3710)	272.97	23	6	29
2	10059	TRPV1 Transport Protein	(3838, 3710)	317.88	232	59	291
3	10075	Bacteriophage MS2	(4096, 4096)	1000*	239	60	299
4	10077	Ribosome (70S)	(4096, 4096)	2198.78	240	60	300
5	10096	Viral Protein	(3838, 3710)	150*	240	60	300
6	10184	Aldolase	(3838, 3710)	150*	236	60	296
7	10240	Lipid Transport Protein	(3838, 3710)	171.72	239	60	299
8	10289	Transport Protein	(3710, 3838)	361.39	240	60	300
9	10291	Transport Protein	(3710, 3838)	361.39	240	60	300
10	10387	Viral Protein	(3710, 3838)	185.87	239	60	299
11	10406	Ribosome (70S)	(3838, 3710)	632.89	191	48	139
12	10444	Membrane Protein	(5760, 4092)	295.89	236	60	296
13	10526	Ribosome (50S)	(7676, 7420)	1085.81	176	44	220
14	10590	TRPV1 Transport Protein	(3710, 3838)	1000*	236	60	296
15	10671	Signaling Protein	(5760, 4092)	77.14	238	60	298
16	10737	Membrane Protein	(5760, 4092)	155.83	233	59	292
17	10760	Membrane Protein	(3838, 3710)	321.69	240	60	300
18	10816	Transport Protein	(7676, 7420)	166.62	240	60	300
19	10852	Signaling Protein	(5760, 4092)	157.81	274	69	343
20	11051	Transcription/DNA/RNA	(3838, 3710)	357.31	240	60	300
21	11057	Hydrolase	(5760, 4092)	149.43	236	59	295
22	11183	Signaling Protein	(5760, 4092)	139.36	240	60	300
		Total			4,948	1,244	6,192

Table S6.
Overview of the test dataset (* indicates the theoretical weight of protein).

SN	EMPIAR ID	Type of Protein	Image Size	Total Structure Weight (kDa)	Number of Images
1	10028	Ribosome (80S)	(4096, 4096)	2135.89	300
2	10081	Transport Protein	(3710, 3838)	298.57	300
3	10345	Signaling Protein	(3838, 3710)	244.68	295
4	11056	Transport Protein	(5760, 4092)	88.94	305
5	10532	Viral Protein	(4096, 4096)	191.76	300
6	10093	Membrane Protein	(3838, 3710)	779.40	295
7	10017	$\boldsymbol{\beta}$ -galactosidase	(4096, 4096)	450*	84
			Total		1,879

Table S7.

Results of three two-stage variants on seven EMPIAR datasets (IDs: 10028, 10081, 10345, 11056, 10532, 10093, 10017). CryoSegNet(Official Weights): U-Net for semantic segmentation followed by SAM post-processing to obtain particle masks. CryoSegNet+ improved U-Net keeps the same pipeline as CryoSegNet but replaces U-Net with our multi-frequency U-Net. CryoSIP: our multi-frequency U-Net for segmentation, with SAM-derived masks fused with the U-Net output via semantic-instance consistency to produce the final particles.

Datasets	Models/Metrics	CryoSegNet (Official Weights)	CryoSegNet+ improved U-Net	CryoSIP
10028	Precision	0.833	0.803	0.846
	Recall	0.944	0.918	0.928
	F1-Score	0.885	0.857	0.885
	Dice Score	0.859	0.848	0.876
10081	Precision	0.835	0.870	0.914
	Recall	0.922	0.913	0.930
	F1-Score	0.876	0.891	0.922
	Dice Score	0.876	0.850	0.918
10345	Precision	0.746	0.733	0.753
	Recall	0.920	0.861	0.882
	F1-Score	0.824	0.792	0.812
	Dice Score	0.743	0.784	0.764
11056	Precision	0.757	0.774	0.789
	Recall	0.687	0.782	0.827
	F1-Score	0.720	0.778	0.809
	Dice Score	0.663	0.748	0.765
10532	Precision	0.796	0.813	0.806
	Recall	0.628	0.645	0.827
	F1-Score	0.702	0.719	0.816
	Dice Score	0.649	0.712	0.785
10093	Precision	0.716	0.753	0.806
	Recall	0.515	0.628	0.724
	F1-Score	0.600	0.685	0.763
	Dice Score	0.537	0.667	0.684
10017	Precision	0.859	0.880	0.871
	Recall	0.616	0.761	0.842
	F1-Score	0.718	0.816	0.856
	Dice Score	0.703	0.811	0.839
Average	Precision	0.792	0.804	0.826
	Recall	0.747	0.787	0.851
	F1-Score	0.761	0.791	0.838
	Dice Score	0.719	0.774	0.804
